# Supplementary material for: Recurring patterns in bacterioplankton dynamics during coastal spring algae blooms
Source: eLife. 2016 Apr 7;5:e11888. doi: 10.7554/eLife.11888 (PMC4829426; doi:10.7554/eLife.11888)
Supplement: Supplementary file 4. — DOI: http://dx.doi.org/10.7554/eLife.11888.014 [file elife-11888-supp4.docx]

| **Probe name** | **Target group** | **Probe sequence (5' ➞ 3')** | **Formamide [%]** | **Reference** |
| --- | --- | --- | --- | --- |
| SAR11-486 | SAR11 clade (*Alphaproteobacteria*) | GGACCTTCTTATTCGGGT | 25 | (Schattenhofer *et al.*, 2009) |
| SAR11-441 | SAR11 clade (*Alphaproteobacteria*) | TACAGTCATTTTCTTCCCCGAC | 25 | (Rappé *et al.*, 2002) |
| ROS537 | *Roseobacter* clade (*Alphaproteobacteria*) | CAACGCTAACCCCCTCC | 35 | (Eilers *et al.*, 2001) |
| GAM42a | *Gammaproteobacteria* | GCCTTCCCACATCGTTT | 35 | (Manz *et al.*, 1992) |
|  | competitor to GAM42a | GCCTTCCCACTTCGTTT |  | (Manz *et al.*, 1992) |
| ALT1413 | *Alteromonas* and *Colwellia* genera (*Gammaproteobacteria*) | TTTGCATCCCACTCCCAT | 40 | (Eilers *et al.*, 2000) |
| SAR92-627 | SAR92 clade (*Gammaproteobacteria*) | CAGACAGTTCTAACTGCAGTTCC | 20 | (Stingl *et al.*, 2007) |
| REI731 | *Reinekea* genus (*Gammaproteobacteria*) | TATCAGCCCAGCAAGTCG | 20 | (Teeling *et al.*, 2012) |
| BAL731 | *Balneatrix* genus (*Gammaproteobacteria*) | TATCAAGCCAGGGCGTCG | 25 | (Teeling *et al.*, 2012) |
| CF319a | *Bacteroidetes* | TGGTCCGTGTCTCAGTAC | 35 | (Manz *et al.*, 1996) |
| POL740 | *Polaribacter* genus (*Bacteroidetes*) | CCCTCAGCGTCAGTACATACGT | 35 | (Malmstrom *et al.*, 2007) |
| FORM181A | *Formosa* genus (*Bacteroidetes*) | GATGCCACTCTAAGAGAC | 25 | (Teeling *et al.*, 2012) |
|  | competitor FORM181A | GATGCCACTCTTAGAGAC |  | (Teeling *et al.*, 2012) |
| FORM181B | *Formosa* sp. Hel1_33_131 (*Bacteroidetes*) | GATGCCACTCTTAGAGAC | 35 | this study |
|  | competitor FORM181B | GATGCCACTCTAAGAGAC |  | this study |
| ULV995 | *Ulvibacter* related clade I (*Bacteroidetes*) | TCCACGCCTGTCAGACTACA | 35 | (Teeling *et al.*, 2012) |
|  | competitor 1 to ULV995 | TCCACTCCTGTCAGACTACA |  | (Teeling *et al.*, 2012) |
|  | competitor 2 to ULV995 | TCCACCCCTGTCAGACTACA |  | (Teeling *et al.*, 2012) |
| NS9-664 | NS9 marine group (*Bacteroidetes*) | ACATGACCTATTCCGCCAACTT | 35 | (Gómez-Pereira *et al.*, 2012) |
| VIS6-814 | genus-level VIS6 clade in the *Cryomorphaceae* (*Bacteroidetes*) | CAGCGAGTGATGATCGTT | 15 | (Gómez-Pereira *et al.*, 2010) |
|  | competitor to VIS6-814 | CAGCGAGTGATCATCGTT |  | (Gómez-Pereira *et al.*, 2010) |
|  | helper 1 to VIS6-814 | TACGGCGTGGACTACCAGGGT |  | this study |
|  | helper 2 to VIS6-814 | CCGCYGACAGTATATCGCCAA |  | this study |
| NS3a-840 | NS3a marine group (*Bacteroidetes*) | CTTAGCCGCTCAGAACTCAAGG | 35 | this study |
|  | competitor 1 to NS3a-840 | CTTGGCCGCCCAGAACTCAAG |  | this study |
|  | competitor 2 to NS3a-840 | CTTGGCCGCCCAGCACTCAAGG |  | this study |
|  | helper 1 to NS3a-840 | TYCCGAACAGCTAGTATCCATCGTT |  | this study |
|  | helper 2 to NS3a-840 | CCAGGTGGGATACTTATCACTTTCG |  | this study |
| NS5/VIS1-575 | VIS1 genus-level clade of the NS5 marine group (*Bacteroidetes*) | CTTAACAAACAGCCTGCGGACC | 35 | (Gómez-Pereira *et al.*, 2010) |
|  | competitor to NS5/VIS1-575 | CTTAAAAAACAGCCTGCGGACC |  | (Gómez-Pereira *et al.*, 2010) |
| CYT-734 | *Marinoscillum* (*Bacteroidetes; Cytophagia*) | CAGTTTCTGCCTAGTAAG | 25 | (Gómez-Pereira *et al.*, 2012) |

**Supplementary file 4.** Specific oligonucleotide probes used for quantification of free-living (0.2 - 3 µm) bacterioplankton populations by fluorescence *in situ* hybridization (FISH).
