# Supplementary material for: Recurring patterns in bacterioplankton dynamics during coastal spring algae blooms
Source: eLife. 2016 Apr 7;5:e11888. doi: 10.7554/eLife.11888 (PMC4829426; doi:10.7554/eLife.11888)
Supplement: Supplementary file 6. — Correlation values were considered only when p-value <0.05. Spearman rank correlations measure the strength of association between two ranked variables. A precondition (assumption) for this nonparametric statistical analysis is that the two variables share a monotonic relationship (i.e. when one variable increases, the other either consistently increases as well or consistently decreases). This monotonic relationship does, however, not need to be linear. While no particularly strong correlations were found between major algae groups and bacterioplankton clades, some noteworthy trends were detected. For example, Ulvibacter abundances were positively correlated with diatoms (in particular centrales) and negatively correlated with silicoflagellates, whereas an opposite trend was observed for the VIS1 clade of the NS5 marine group (i.e. negative correlation with diatoms and a positive correlation with silicoflagellates). While the explanatory power of such pairwise rank-based correlations is limited and correlation does not necessarily imply causation, these data suggest at least for Ulvibacter sp. (and to a lesser extent for Formosa clade B) that they are associated with diatoms, while the NS3a, NS5 and NS9 marine groups seemed to be rather positively correlated with flagellates such as dino- and silicoflagellates. The positive correlation between Ulvibacter sp. and diatoms was supported by Spearman rank correlation analysis between the abundances of prominent bacterioplankton clades and distinct phytoplankton groups. Ulvibacter abundances were positively correlated with the diatoms Mediopyxis helysia, Chaetoceros debilis, Chaetoceros minimus, Thalassiosira nordenskioeldii and the Phaeocystis spp. haptophytes. Correlations of other bacterioplankton groups with distinct phytoplankton groups were less pronounced. Noteworthy positive correlations were found between Chattonella spp. and Bacteroidetes abundances (probe CF319a), in particular Marinoscillum spp. (probe CYT-73 [file elife-11888-supp6.docx]

**Spearman rank correlations of abundant *Bacteroidetes* clades to phytoplankton groups. Correlation values were considered only when p-value <0.05.**

|  | Diatoms | Pennales | Centrales | Greenalgae | Dinoflagellates | Silicoflagellates | Coccolithophorids | Flagellates | Ciliates |
| --- | --- | --- | --- | --- | --- | --- | --- | --- | --- |
| CF319a |  |  | 0.266 |  | 0.456 |  | 0.268 | 0.315 | 0.195 |
| p-value: |  |  | 0.002 |  | <0.001 |  | 0.002 | <0.001 | 0.024 |
| POL740 |  | -0.203 |  |  | 0.406 |  |  | 0.229 | 0.173 |
| p-value: |  | 0.019 |  |  | <0.001 |  |  | 0.008 | 0.046 |
| FORM 181A |  | -0.314 |  |  | 0.249 | -0.266 |  |  |  |
| p-value: |  | <0.001 |  |  | 0.005 | 0.003 |  |  |  |
| FORM181B | 0.228 |  | 0.217 | 0.198 | 0.270 |  |  | 0.236 |  |
| p-value: | 0.010 |  | 0.014 | 0.025 | 0.002 |  |  | 0.007 |  |
| ULV995 | 0.515 | 0.202 | 0.661 |  | 0.288 | -0.291 | 0.240 | 0.296 |  |
| p-value: | <0.001 | 0.021 | <0.001 |  | <0.001 | <0.001 | 0.060 | <0.001 |  |
| VIS6-814 | 0.212 |  | 0.400 |  | 0.177 |  |  |  | 0.178 |
| p-value: | 0.014 |  | <0.001 |  | 0.042 |  |  |  | 0.040 |
| NS3a- 840 |  |  |  | -0.179 | 0.443 | 0.289 |  | 0.244 | 0.221 |
| p-value: |  |  |  | 0.042 | <0.001 | <0.001 |  | 0.005 | 0.012 |
| NS5/VIS1-575 | -0.458 | -0.202 | -0.540 |  |  | 0.463 |  |  |  |
| p-value: | <0.001 | 0.021 | <0.001 |  |  | <0.001 |  |  |  |
| NS9-664 | -0.261 | -0.419 |  | -0.392 | 0.347 |  | 0.301 | 0.233 |  |
| p-value: | 0.002 | <0.001 |  | <0.001 | <0.001 |  | <0.001 | 0.007 |  |
| CYT-734 |  | -0.188 |  | -0.256 | 0.475 |  |  | 0.366 |  |
| p-value: |  | 0.034 |  | 0.004 | <0.001 |  |  | <0.001 |  |

**Supplementary file 6.**

Spearman rank correlations measure the strength of association between two ranked variables. A precondition (assumption) for this nonparametric statistical analysis is that the two variables share a monotonic relationship (i.e. when one variable increases, the other either consistently increases as well or consistently decreases). This monotonic relationship does, however, not need to be linear.

While no particularly strong correlations were found between major algae groups and bacterioplankton clades, some noteworthy trends were detected. For example, *Ulvibacter* abundances were positively correlated with diatoms (in particular centrales) and negatively correlated with silicoflagellates, whereas an opposite trend was observed for the VIS1 clade of the NS5 marine group (i.e. negative correlation with diatoms and a positive correlation with silicoflagellates). While the explanatory power of such pairwise rank-based correlations is limited and correlation does not necessarily imply causation, these data suggest at least for *Ulvibacter* sp. (and to a lesser extent for *Formosa* clade B) that they are associated with diatoms, while the NS3a, NS5 and NS9 marine groups seemed to be rather positively correlated with flagellates such as dino- and silicoflagellates.

1

**Spearman rank correlations of abundant *Bacteroidetes* clades to specific phytoplankton groups. Correlation values were considered only when p-value <0.05.**

|  | *Mediopyxis helysia* | *Chaetoceros*  *debilis* | *Chaetoceros minimus* | *Rhizosolenia styliformis* | *Thalassiosira nordenskioeldii* | *Phaeocystis*  sp. | *Chattonella*  sp. |
| --- | --- | --- | --- | --- | --- | --- | --- |
| CF319a |  | 0.264 | 0.246 |  |  | 0.341 | 0.350 |
| p-value: |  | 0.002 | 0.004 |  |  | <0.001 | <0.001 |
| POL740 |  |  |  |  |  | 0.201 | 0.380 |
| p-value: |  |  |  |  |  | 0.021 | <0.001 |
| FORM 181A |  |  | 0.264 |  | -0.226 |  |  |
| p-value: |  |  | 0.003 |  | 0.010 |  |  |
| FORM181B | 0.302 |  |  | 0.272 | 0.225 | 0.283 | 0.189 |
| p-value: | <0.001 |  |  | 0.002 | 0.011 | 0.001 | 0.033 |
| ULV995 | 0.361 | 0.479 | 0.388 |  | 0.365 | 0.496 |  |
| p-value: | <0.001 | <0.001 | <0.001 |  | <0.001 | <0.001 |  |
| VIS6-814 |  | 0.228 | 0.224 |  |  | 0.208 |  |
| p-value: |  | 0.008 | 0.010 |  |  | 0.016 |  |
| NS3a- 840 |  |  |  |  | -0.177 |  | 0.345 |
| p-value: |  |  |  |  | 0.044 |  | <0.001 |
| NS5/VS1-575 |  | -0.354 | -0.441 |  | -0.295 | -0.256 | 0.369 |
| p-value: |  | <0.001 | <0.001 |  | <0.001 | 0.003 | <0.001 |
| NS9-664 | -0.188 |  | 0.283 | 0.231 |  |  | 0.285 |
| p-value: | 0.033 |  | <0.001 | 0.008 |  |  | <0.001 |
| CYT-734 |  |  |  |  |  |  | 0.425 |
| p-value: |  |  |  |  |  |  | <0.001 |

The positive correlation between *Ulvibacter* sp. and diatoms was supported by Spearman rank correlation analysis between the abundances of prominent bacterioplankton clades and distinct phytoplankton groups. *Ulvibacter* abundances were positively correlated with the diatoms *Mediopyxis helysia, Chaetoceros debilis, Chaetoceros minimus, Thalassiosira nordenskioeldii* and the *Phaeocystis* spp. haptophytes. Correlations of other bacterioplankton groups with distinct phytoplankton groups were less pronounced. Noteworthy positive correlations were found between *Chattonella* spp. and *Bacteroidetes* abundances (probe CF319a), in particular *Marinoscillum* spp. (probe CYT-734), *Polaribacter* spp. (probe POL740) and the NS5/VIS1 and NS3a marine groups. The NS5/VIS1 marine group was negatively correlated with most diatom groups and the *Phaeocystis* sp. haptophytes, and only positively correlated with *Chattonella* species. This might indicate a preference for *Chattonella* spp. or simply reflect that the members of the VIS1 clade of the NS5 marine group were less competitive than other *Flavobacteriia* under the conditions where other algae dominated.

2

**Spearman rank correlations of abundant *Bacteroidetes* clades to physicochemical parameters. Correlation values were considered only when p-value <0.05.**

|  | Temperature | Salinity | Chlorophyll *a* | Silicate | Phosphate | Nitrite | Nitrate | Ammonium |
| --- | --- | --- | --- | --- | --- | --- | --- | --- |
| CF319a | 0.771 |  | 0.334 | -0.497 | -0.583 | -0.487 | -0.457 | -0.193 |
| p-value: | <0.001 |  | <0.001 | <0.001 | <0.001 | <0.001 | <0.001 | 0.026 |
| POL740 | 0.629 |  | 0.201 | -0.238 | -0.367 | -0.450 | -0.310 |  |
| p-value: | <0.001 |  | 0.022 | 0.006 | <0.001 | <0.001 | <0.001 |  |
| FORM 181A | 0.617 |  |  | -0.430 | -0.467 | -0.512 | -0.426 |  |
| p-value: | <0.001 |  |  | <0.001 | <0.001 | <0.001 | <0.001 |  |
| FORM181B | 0.273 | -0.326 | 0.296 | -0.551 | -0.425 |  |  | -0.358 |
| p-value: | 0.002 | <0.001 | <0.001 | <0.001 | <0.001 |  |  | <0.001 |
| ULV995 | 0.451 |  | 0.453 | -0.861 | -0.763 |  | -0.518 | -0.446 |
| p-value: | <0.001 |  | <0.001 | <0.001 | <0.001 |  | <0.001 | <0.001 |
| VIS6-814 | 0.500 |  | 0.251 | -0.456 | -0.474 | -0.401 | -0.393 |  |
| p-value: | <0.001 |  | 0.004 | <0.001 | <0.001 | <0.001 | <0.001 |  |
| NS3a- 840 | 0.350 | -0.429 |  | 0.212 |  |  | 0.218 | 0.225 |
| p-value: | <0.001 | <0.001 |  | 0.015 |  |  | 0.012 | 0.010 |
| NS5/VIS1-575 |  | -0.206 |  | 0.578 | 0.439 |  | 0.453 | 0.431 |
| p-value: |  | 0.018 |  | <0.001 | <0.001 |  | <0.001 | <0.001 |
| NS9-664 | 0.536 |  |  |  | -0.329 | -0.364 | -0.294 |  |
| p-value: | <0.001 |  |  |  | <0.001 | <0.001 | <0.001 |  |
| CYT-734 | 0.558 | -0.282 |  |  | -0.240 | -0.305 |  |  |
| p-value: | <0.001 | 0.001 |  |  | 0.006 | <0.001 |  |  |

Spearman rank correlations between major bacterioplankton clades and physicochemical parameters were most conclusive for *Ulvibacter* species. *Ulvibacter* abundances were positively correlated with chlorophyll *a*, consistent with the previously detected positive correlation with in particular diatoms. *Ulvibacter* abundances were also negatively correlated with silicate, phosphate, nitrate and ammonium. In particular the strong negative correlation with silicate, which is the limiting factor for diatom frustule formation, supports a particularly strong association of *Ulvibacter* spp. and diatoms. Similar but weaker trends were observed also for other bacterioplankton clades, such as *Polaribacter, Formosa* clade B, or VIS6 clade *Cryomorphaceae.* Of all genus-level clades, *Polaribacter* and *Formosa* clade A showed the strongest associations with temperature.

3
