# Supplementary material for: Recurring patterns in bacterioplankton dynamics during coastal spring algae blooms
Source: eLife. 2016 Apr 7;5:e11888. doi: 10.7554/eLife.11888 (PMC4829426; doi:10.7554/eLife.11888)
Supplement: Supplementary file 7. — For all tested clades of Flavobacteriia, multiple abiotic factors and multiple algae groups were obtained as explanatory variables. The strongest abiotic predictors were temperature, salinity, silicate and nitrate. The strongest biotic predictors were Phaeocystis spp. haptophytes, Rhizosolenia spp., Chaetoceros debilis, and Chaetoceros minimus diatoms and the silicoflagellate Chattonella. It should be noted though that these regressions were computed based on log-transformed abundance data and not algae biovolumes (which were not measured). Insofar the in influence of the rather small cell-sized algae such as Chaetoceros miniumus is likely overestimated. Such limitations notwithstanding it is noteworthy that in no case a simple one-to-one relationship between specific algae and specific bacterioplankton groups was obtained so far. DOI: http://dx.doi.org/10.7554/eLife.11888.017 [file elife-11888-supp7.docx]

**Linear regression analysis**

**- Total cell counts (March-May 2009-2012) -**

independent variables:

Temperature, Salinity, Silicate, Phosphate, Nitrite, Nitrate, Ammonium, Greenalgae, Silicoflagellates, Coccolithophorids, Flagellates, Ciliates, *Mediopyxis*, *Chaetoceros debilis*, *Chaetoceros minimus*, *Rhizosolenia*, *Thalassosira*, *Dinophyceae*, *Phaeocystis*, *Chattonella*

The dependent variable total cell count (TCC) can be predicted from a linear combination of the independent variables:

Summary Table

| Step # | Variables Entered | R | RSqr | P |
| --- | --- | --- | --- | --- |
| 1 | Temperature | 0.64 | 0.41 | <0.001 |
| 2 | *Phaeocystis* | 0.724 | 0.525 | <0.001 |
| 3 | Flagellates | 0.777 | 0.603 | <0.001 |
| 4 | *Mediopyxis helysia* | 0.798 | 0.636 | <0.001 |
| 5 | *Chattonella* | 0.809 | 0.655 | 0.013 |

**Supplementary file 7.**

Linear regression analyses were computed in order to test, whether the abundances of major clades of *Flavobacteriia* were influenced by abiotic factors or by abundant algae groups. For all tested clades of *Flavobacteriia,* multiple abiotic factors and multiple algae groups were obtained as explanatory variables. The strongest abiotic predictors were temperature, salinity, silicate and nitrate. The strongest biotic predictors were *Phaeocystis* spp. haptophytes, *Rhizosolenia* spp., *Chaetoceros debilis,* and *Chaetoceros minimus* diatoms and the silicoflagellate *Chattonella.* It should be noted though that these regressions were computed based on log-transformed abundance data and not algae biovolumes (which were not measured). Insofar the influence of the rather small cell- sized algae such as *Chaetoceros minimus* is likely overestimated. Such limitations notwithstanding it is noteworthy that in no case a simple one-to-one relationship between specific algae and specific bacterioplankton groups was obtained.


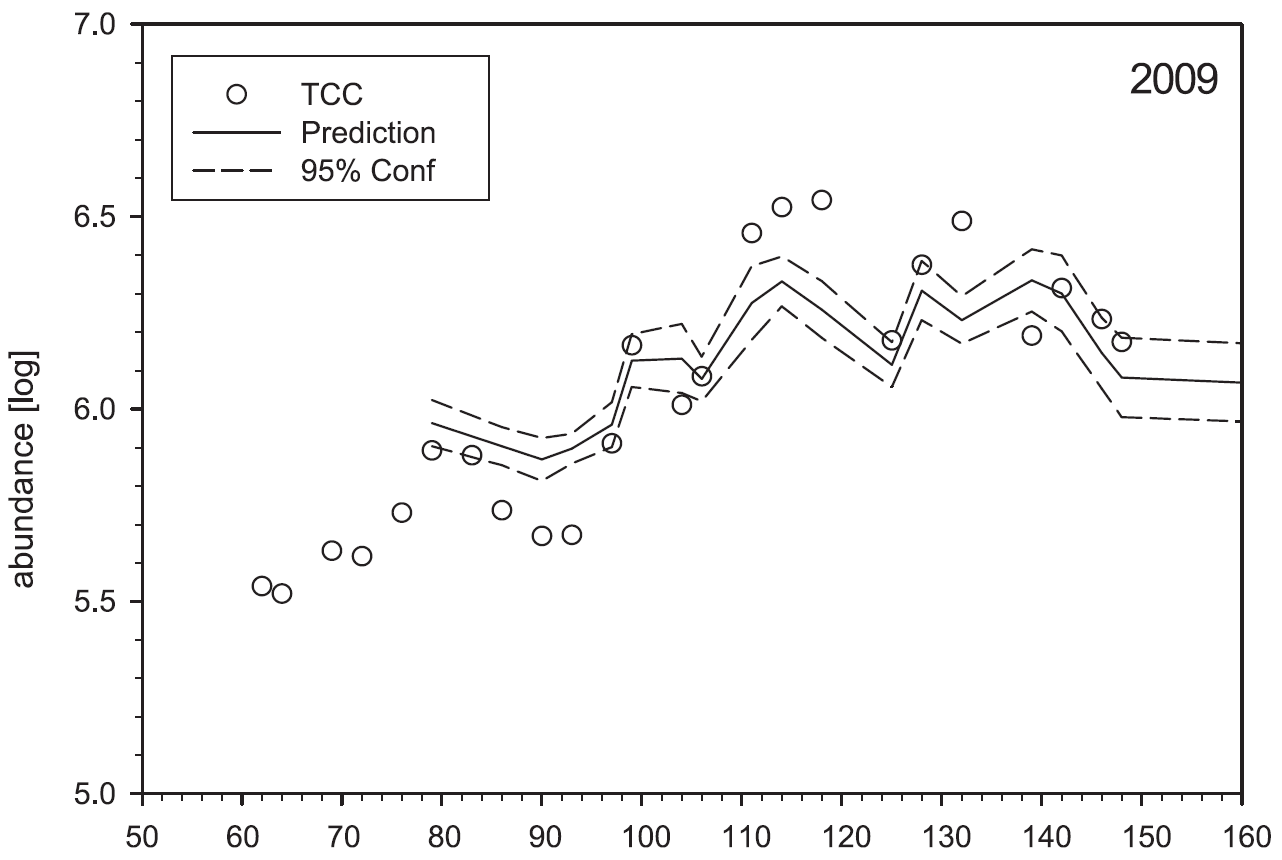


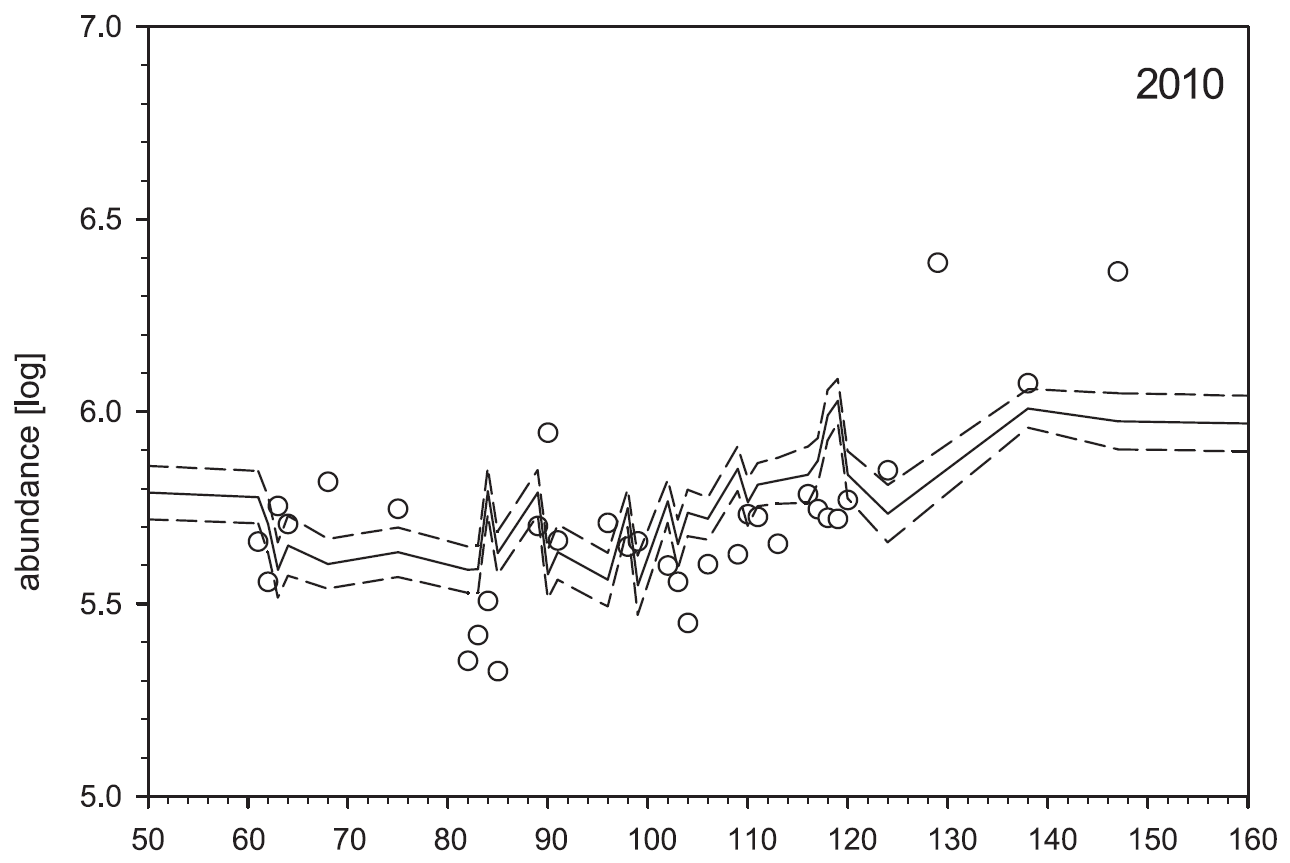


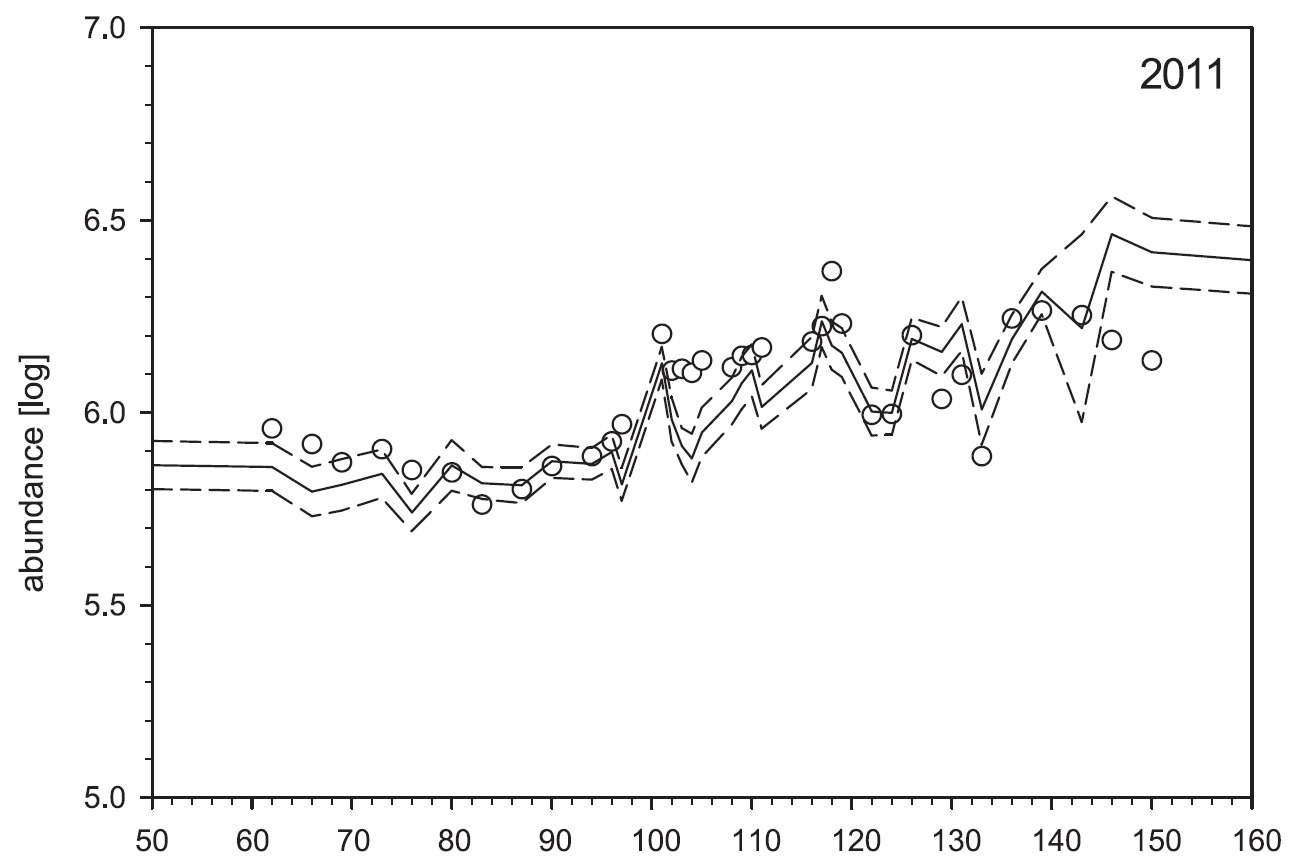


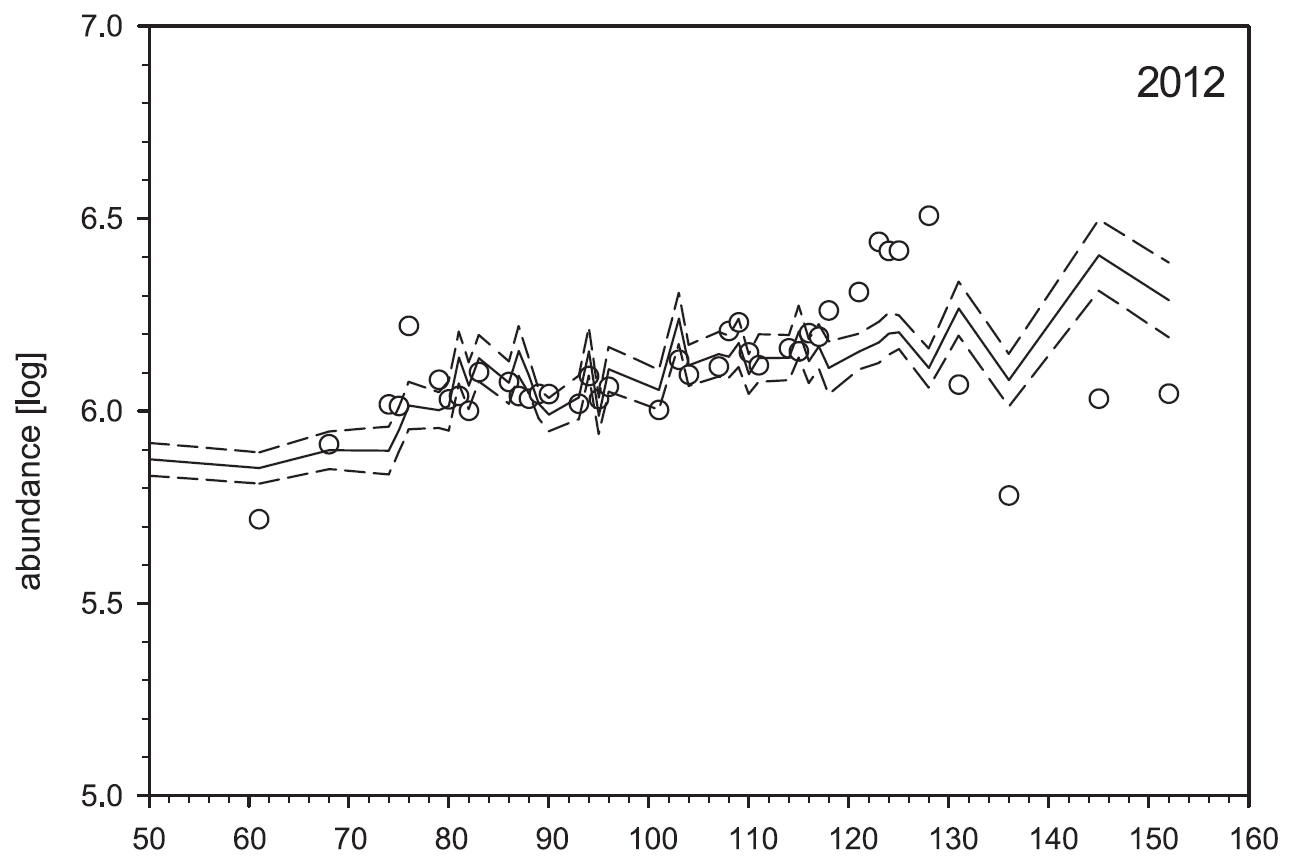


Julian day

**Linear regression analysis**

**- CF319a/*Bacteroidetes* (March-May 2009-2012) -**

independent variables:

Temperature, Salinity, Silicate, Phosphate, Nitrite, Nitrate, Ammonium, Greenalgae, Silicoflagellates, Coccolithophorids, Flagellates, Ciliates, *Mediopyxis*, *Chaetoceros debilis*, *Chaetoceros minimus*, *Rhizosolenia*, *Thalassosira*, *Dinophyceae*, *Phaeocystis*, *Chattonella*

The dependent variable CF319a can be predicted from a linear combination of the independent variables:

Summary Table

| Step # | Variables Entered | R | RSqr | P |
| --- | --- | --- | --- | --- |
| 1 | Temperature | 0.719 | 0.517 | <0.001 |
| 2 | Nitrate | 0.765 | 0.585 | <0.001 |
| 3 | *Chaet. debilis* | 0.786 | 0.618 | <0.001 |
| 4 | *Phaeocystis* | 0.824 | 0.679 | <0.001 |
| 5 | *Chattonella* | 0.84 | 0.705 | <0.001 |
| 6 | Ammonium | 0.849 | 0.721 | 0.01 |


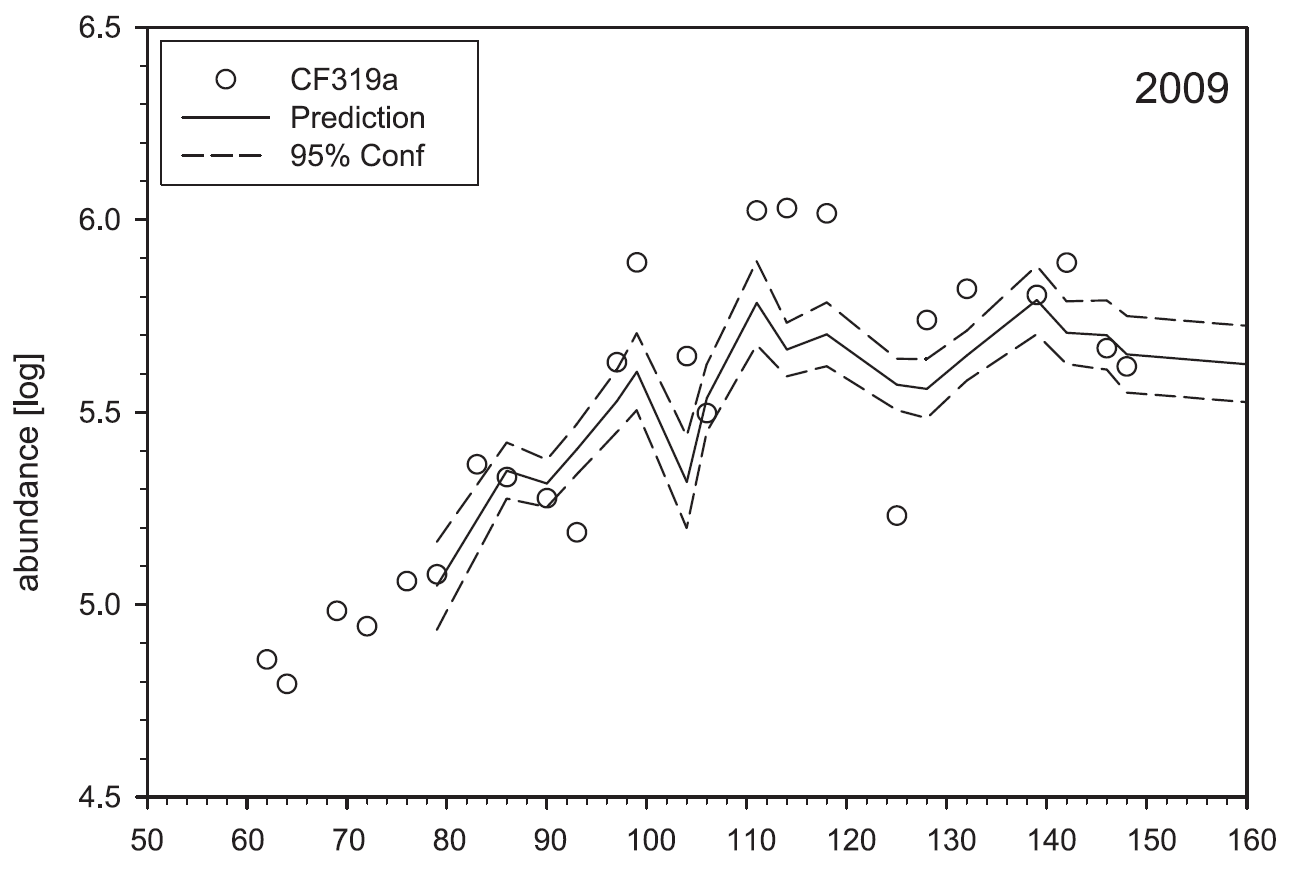


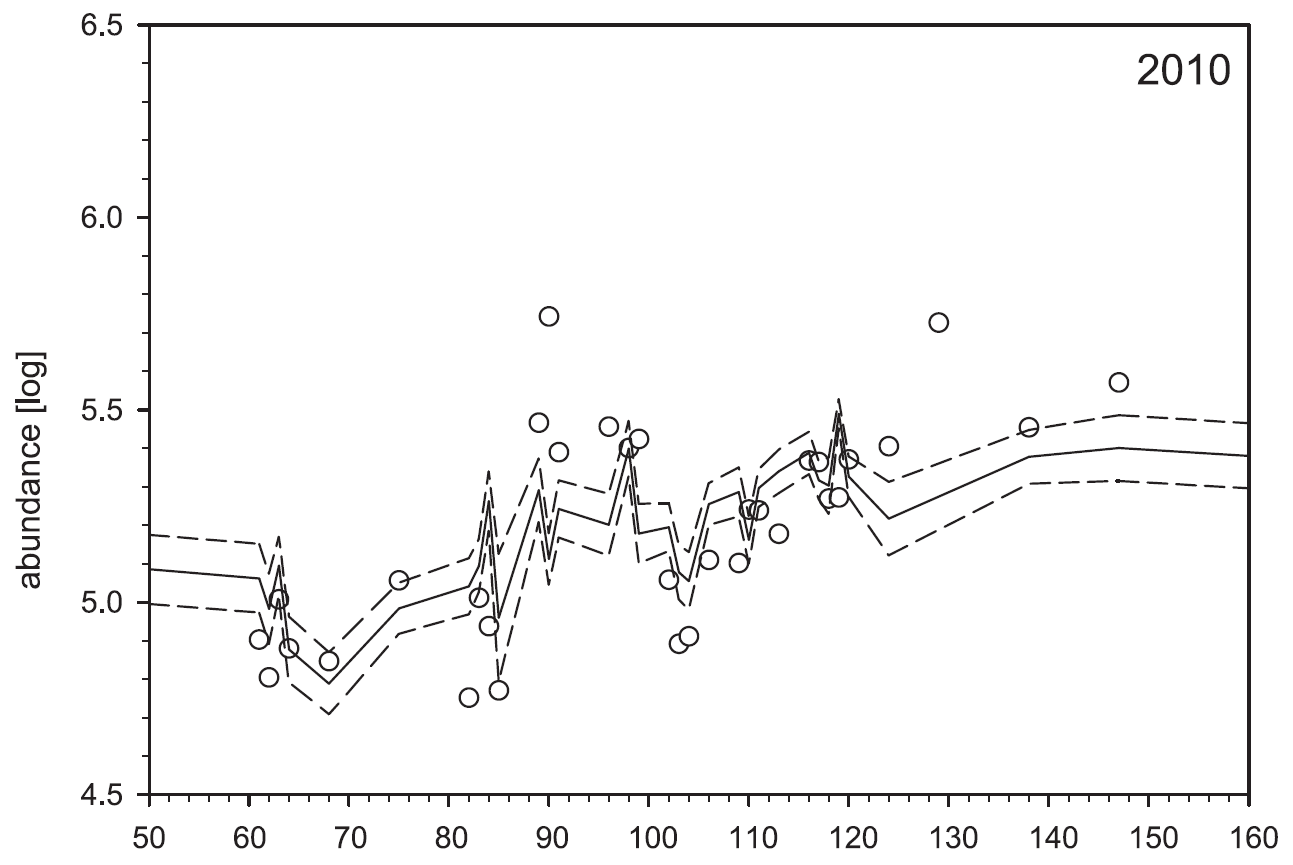


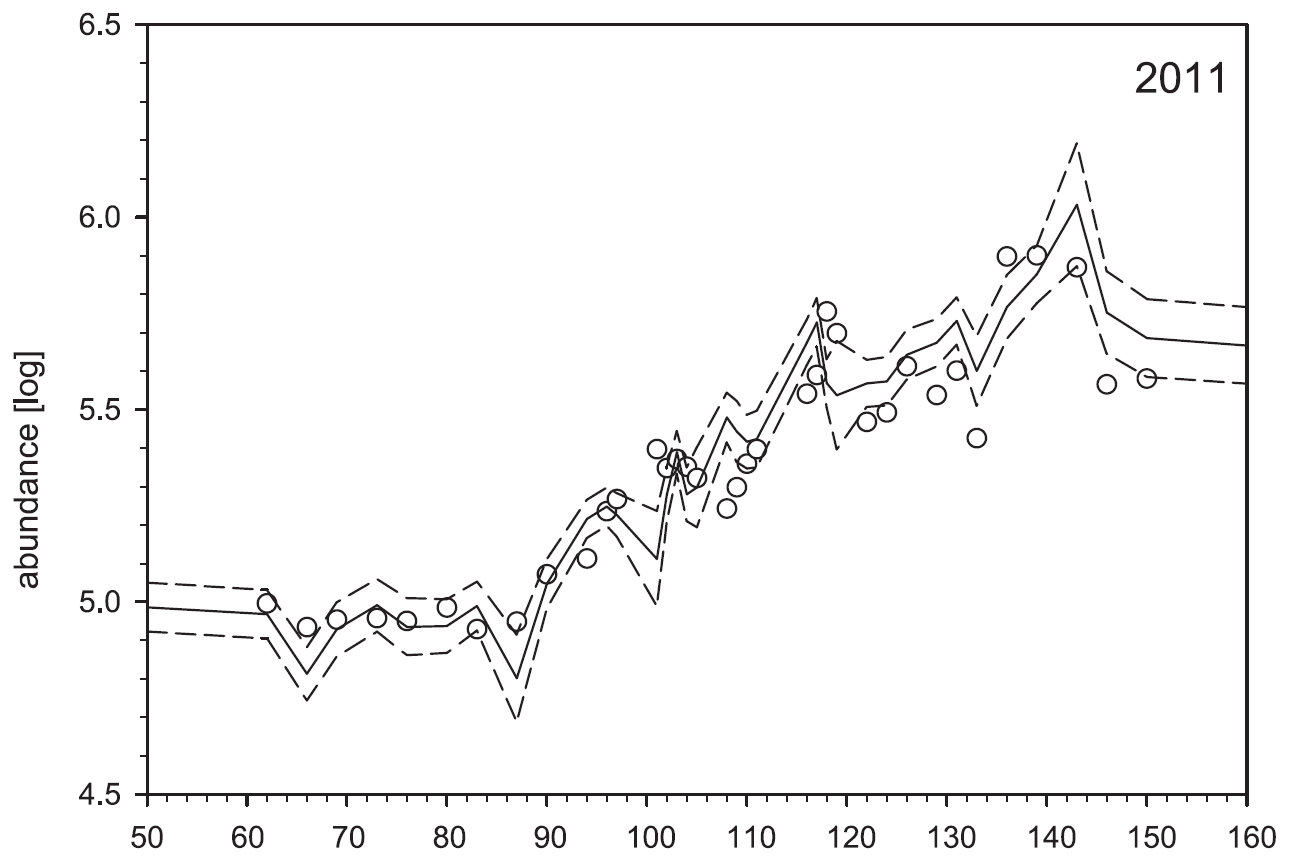


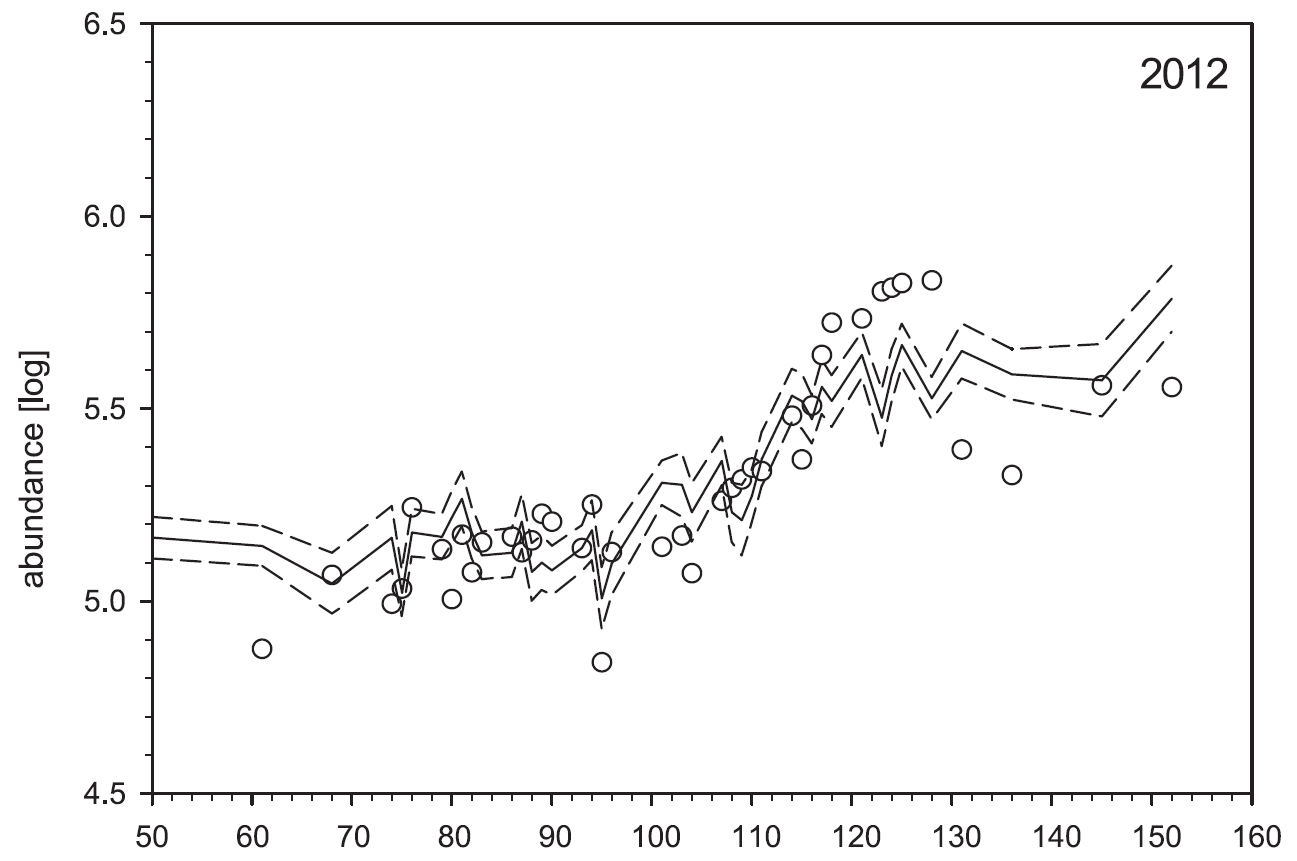


Julian day

**Linear regression analysis**

**- *Polaribacter* (March-May 2009-2012) -**

independent variables:

Temperature, Salinity, Silicate, Phosphate, Nitrite, Nitrate, Ammonium, Greenalgae, Silicoflagellates, Coccolithophorids, Flagellates, Ciliates, *Mediopyxis, Chaetoceros debilis, Chaetoceros minimus, Rhizosolenia, Thalassosira, Dinophyceae, Phaeocystis, Chattonella*

The dependent variable *Polaribacter* can be predicted from a linear combination of the independent variables:

Summary Table

| Step # | Variables Entered | R | RSqr | P |
| --- | --- | --- | --- | --- |
| 1 | Temperature | 0.538 | 0.289 | <0.001 |
| 2 | Nitrate | 0.572 | 0.327 | <0.001 |
| 3 | *Chattonella* | 0.621 | 0.386 | <0.001 |
| 4 | *Phaeocystis* | 0.674 | 0.455 | <0.001 |
| 5 | Flagellates | 0.69 | 0.477 | 0.011 |
| 6 | *Rhizosolenia styliformis* | 0.705 | 0.497 | 0.004 |
| 7 | *Chaetoceros debilis* | 0.725 | 0.526 | 0.008 |


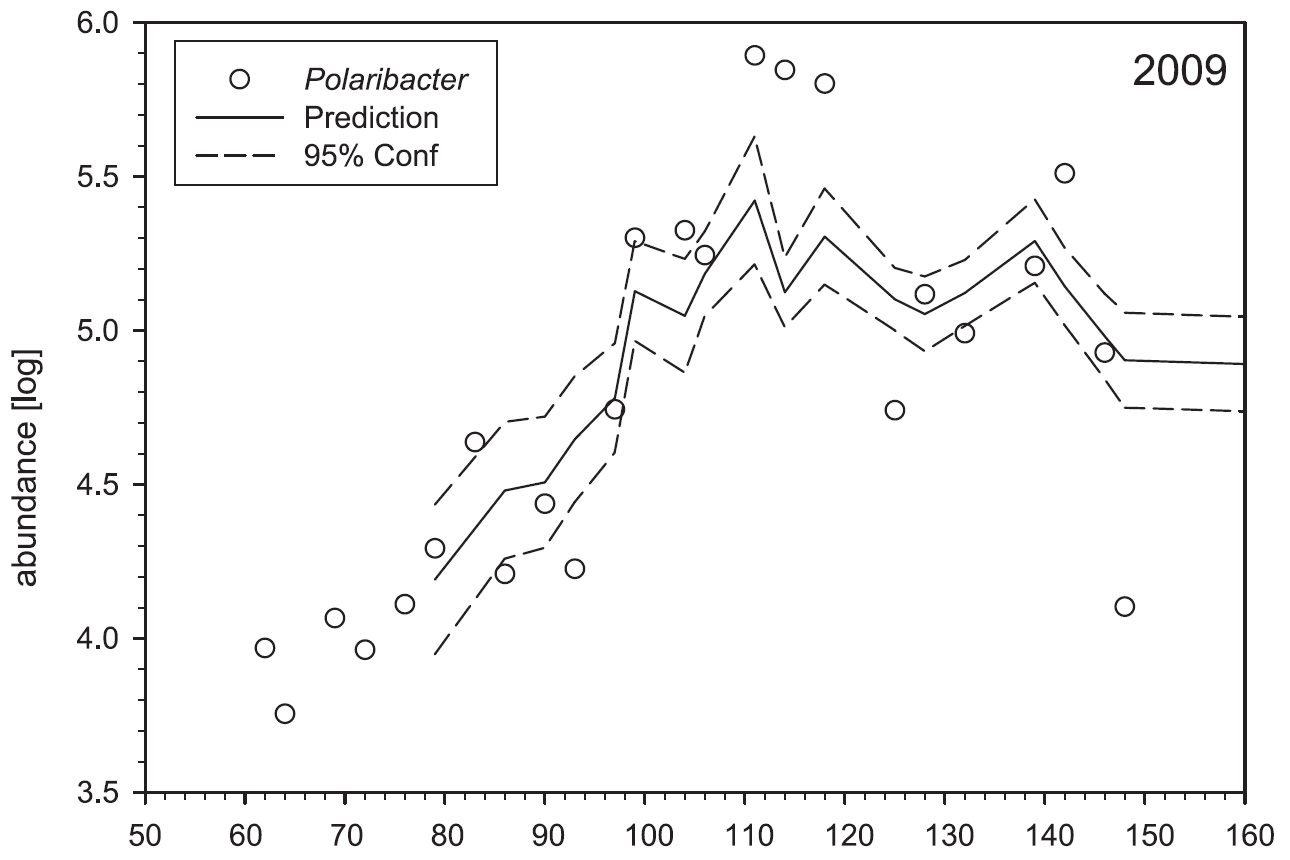


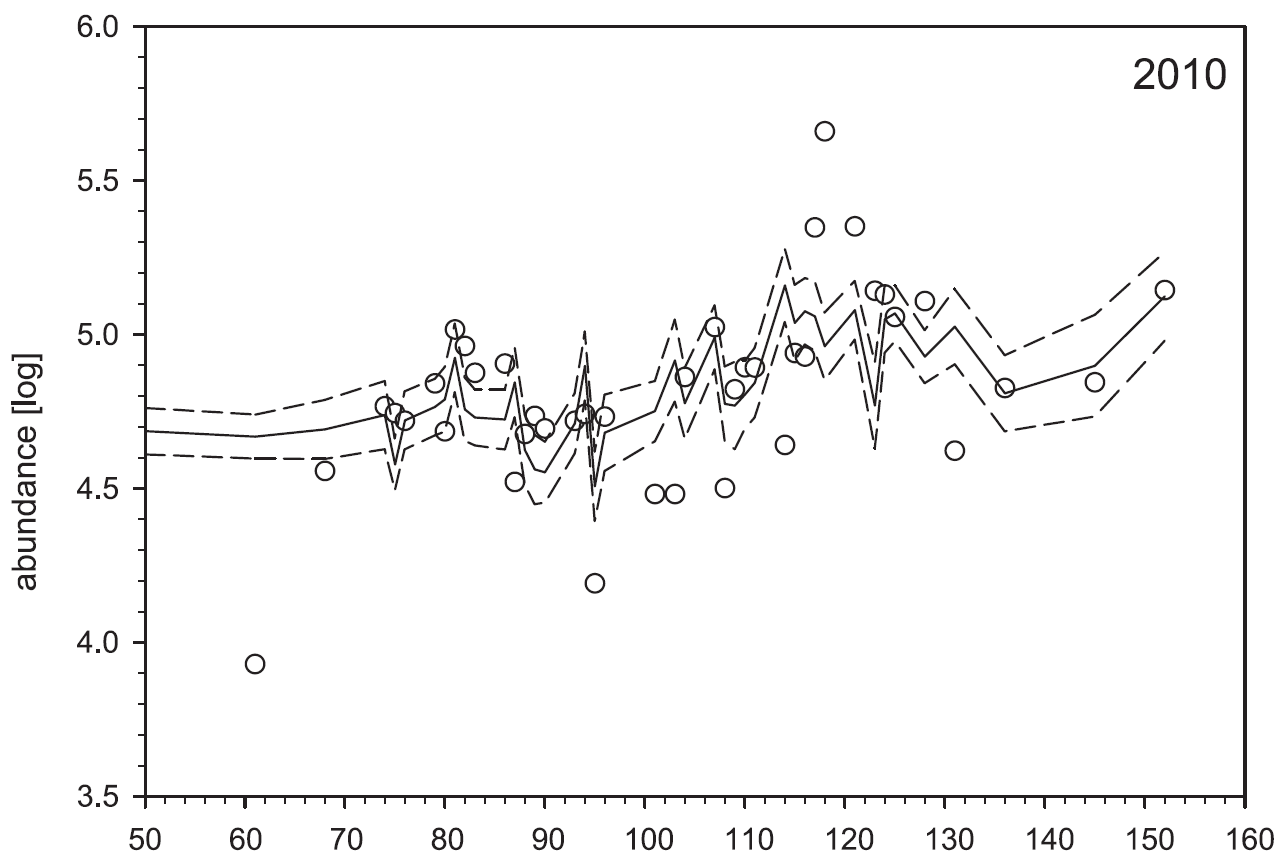


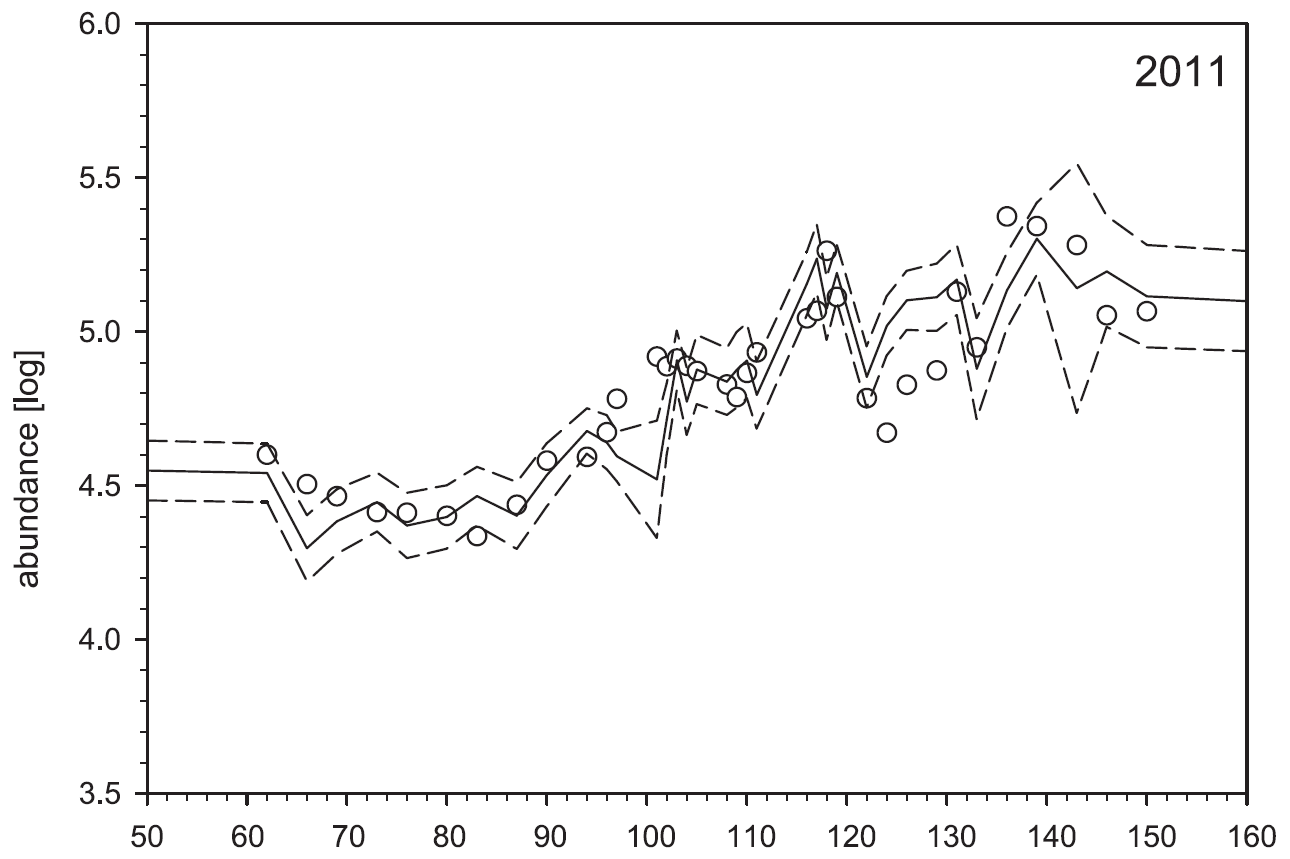


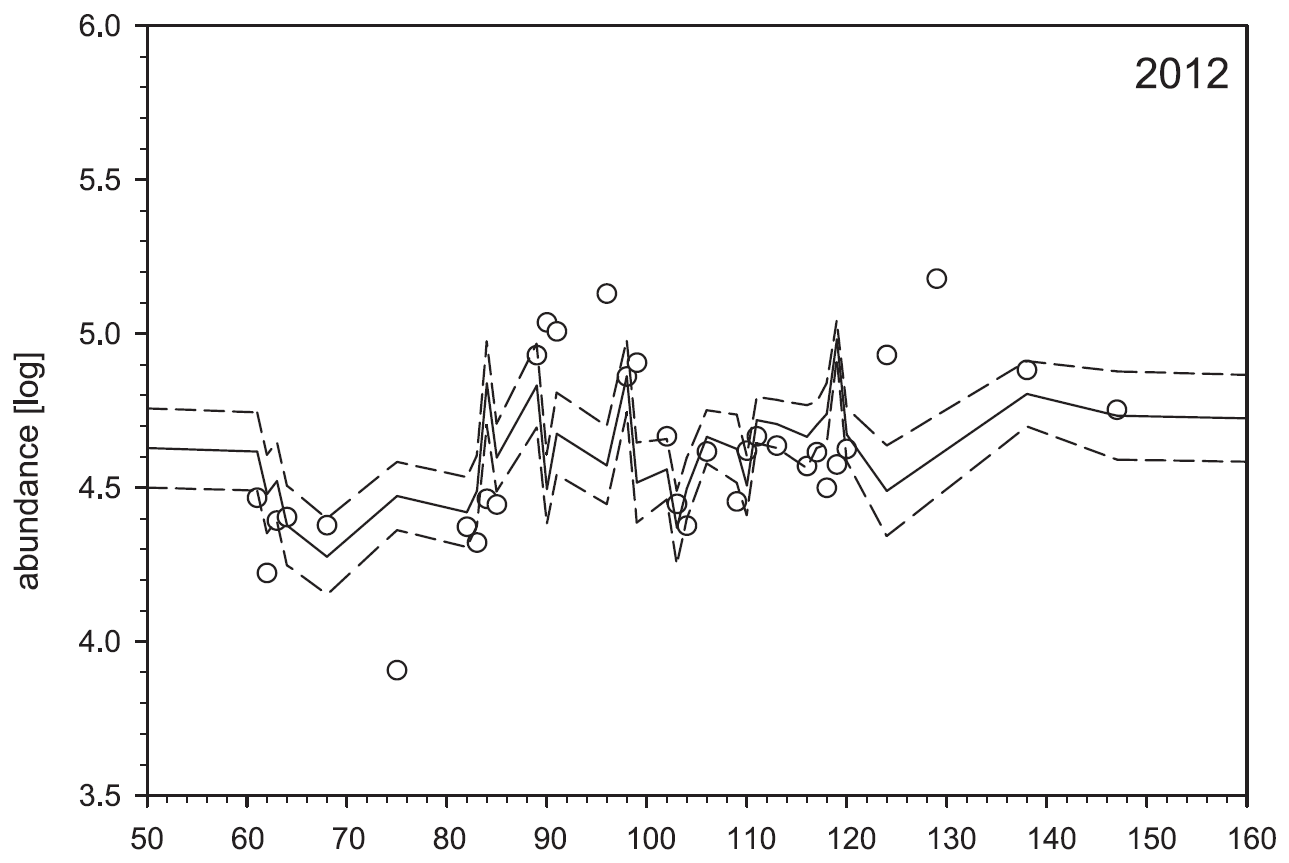


Julian day

**Linear regression analysis**

**- *Formosa* clade A (March-May 2009-2012) -**

independent variables:

Temperature, Salinity, Silicate, Phosphate, Nitrite, Nitrate, Ammonium, Greenalgae, Silicoflagellates, Coccolithophorids, Flagellates, Ciliates, *Mediopyxis, Chaetoceros debilis, Chaetoceros minimus, Rhizosolenia, Thalassosira, Dinophyceae, Phaeocystis, Chattonella*

The dependent variable *Formosa* clade A can be predicted from a linear combination of the independent variables:

Summary Table

| Step # | Variables Entered | R | RSqr | P |
| --- | --- | --- | --- | --- |
| 1 | Temperature | 0.533 | 0.284 | <0.001 |
| 2 | Silicoflagellates | 0.687 | 0.472 | 0.004 |
| 3 | Salinity | 0.722 | 0.522 | 0.004 |
| 4 | Phaeocystis | 0.738 | 0.545 | 0.001 |
| 5 | *Rhizosolenia styliformis* | 0.753 | 0.568 | 0.026 |
| 6 | Silicate | 0.764 | 0.583 | 0.042 |


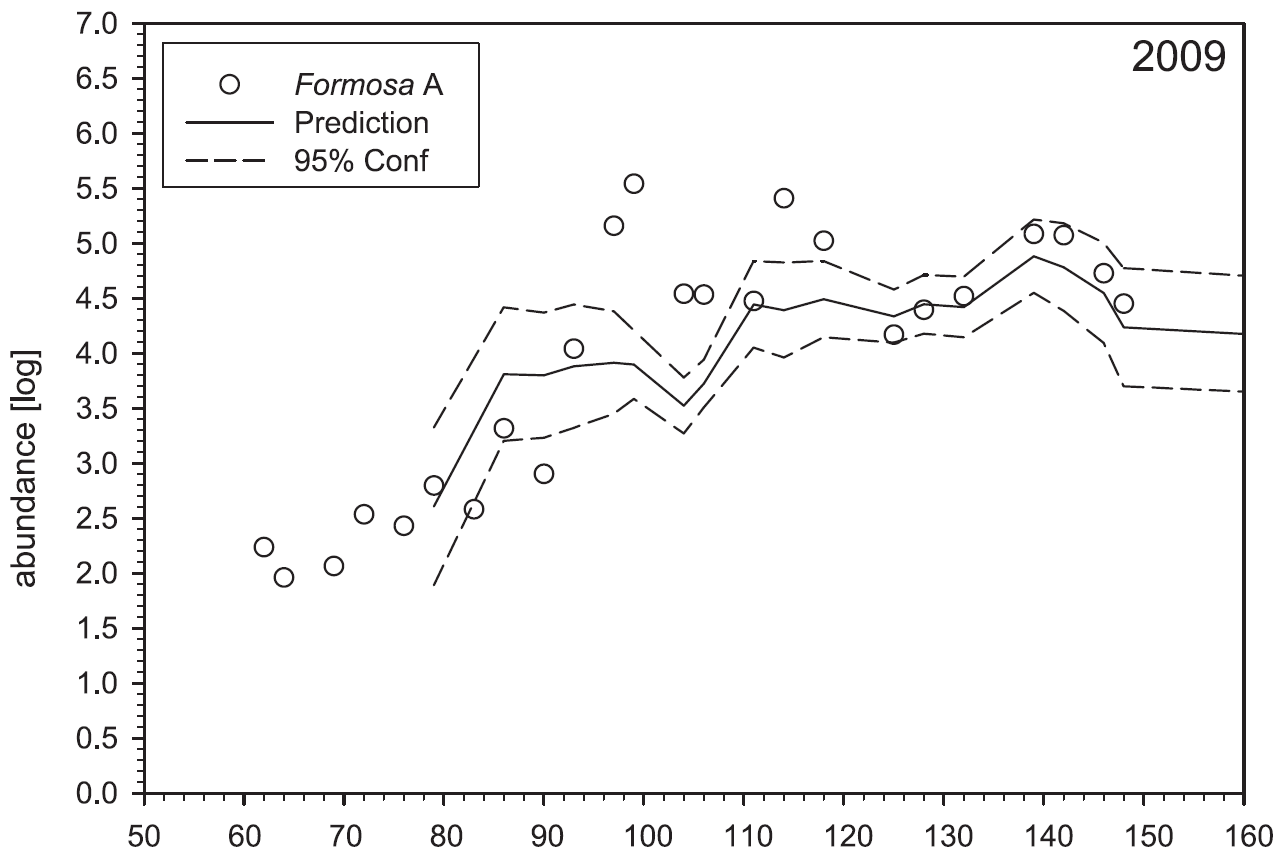


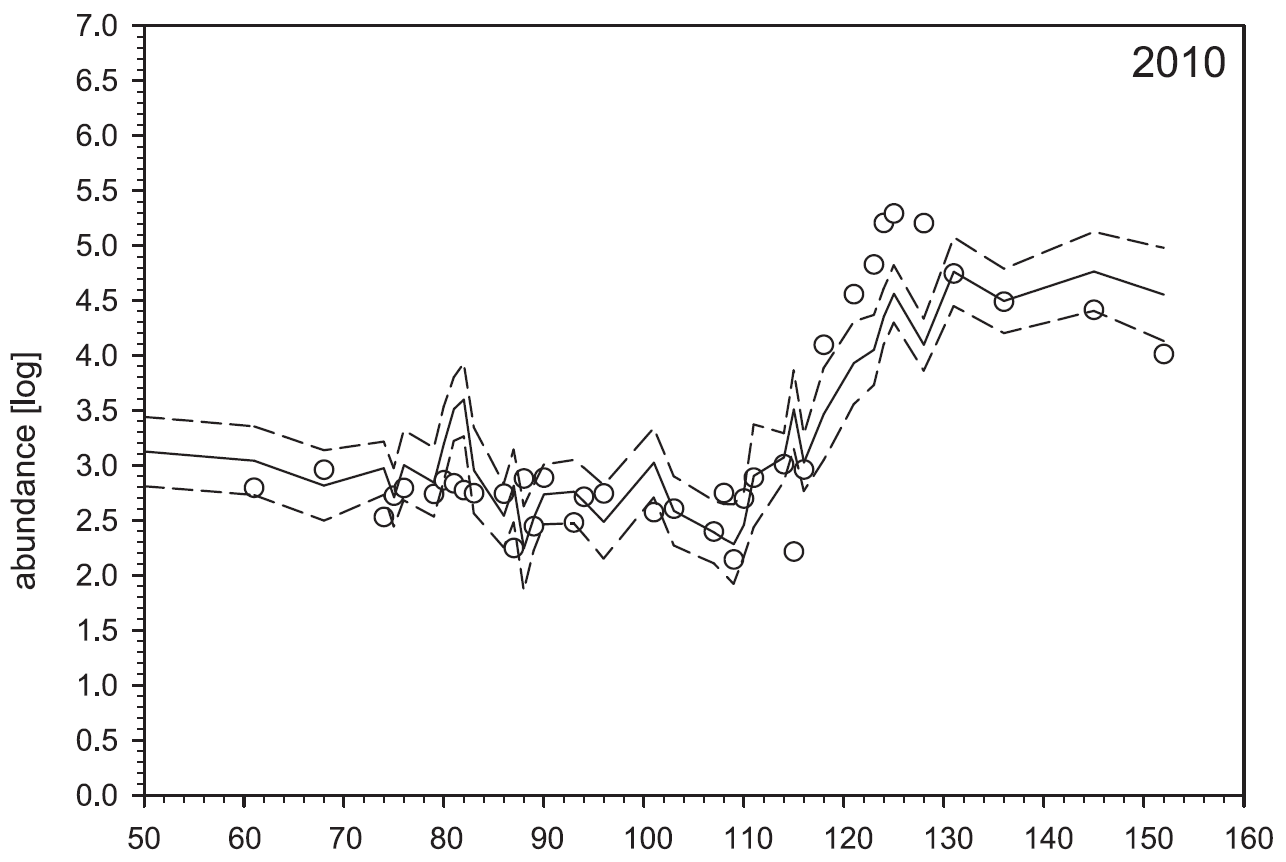


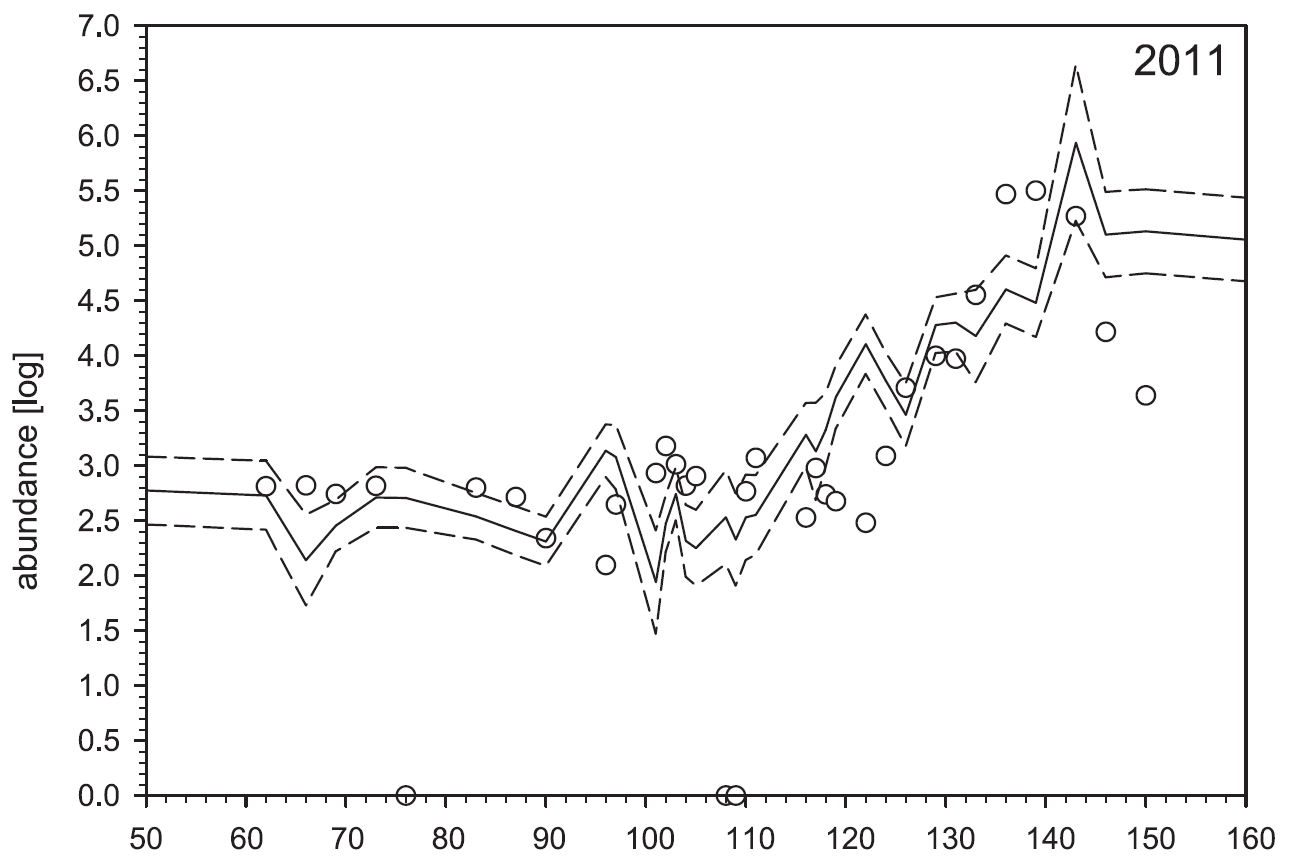


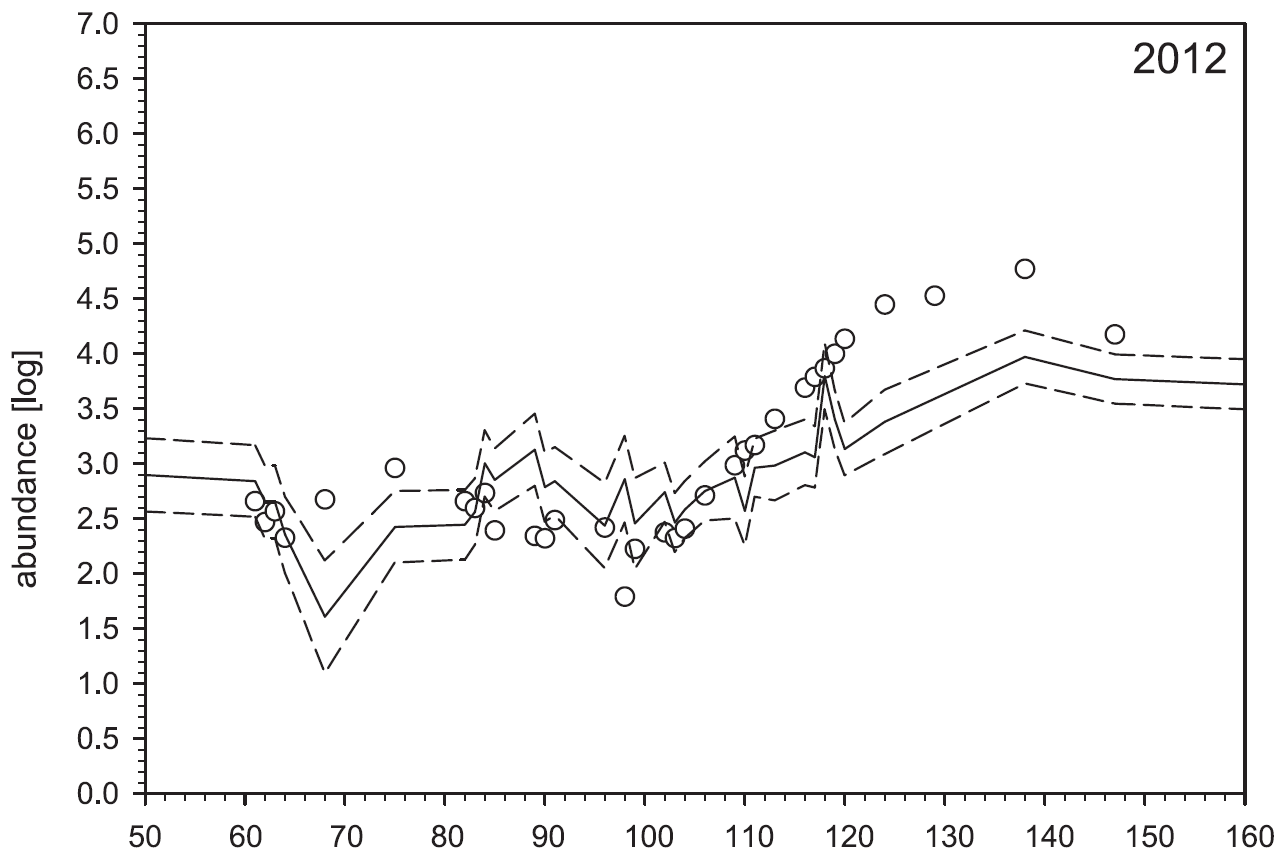


Julian day

**Linear regression analysis**

**- NS9 marine group (March-May 2009-2012) -**

independent variables:

Temperature, Salinity, Silicate, Phosphate, Nitrite, Nitrate, Ammonium, Greenalgae, Silicoflagellates, Coccolithophorids, Flagellates, Ciliates, *Mediopyxis, Chaetoceros debilis, Chaetoceros minimus, Rhizosolenia, Thalassosira, Dinophyceae, Phaeocystis, Chattonella*

The dependent variable NS9 can be predicted from a linear combination of the independent variables:

Summary Table

| Step # | Variables Entered | R | RSqr | P |
| --- | --- | --- | --- | --- |
| 1 | Temperature | 0.475 | 0.225 | <0.001 |
| 2 | *Mediopyxis helysia* | 0.606 | 0.367 | <0.001 |
| 3 | Greenalgae | 0.666 | 0.444 | <0.001 |
| 4 | Flagellates | 0.689 | 0.475 | <0.001 |
| 5 | *Phaeocystis* | 0.717 | 0.515 | 0.002 |


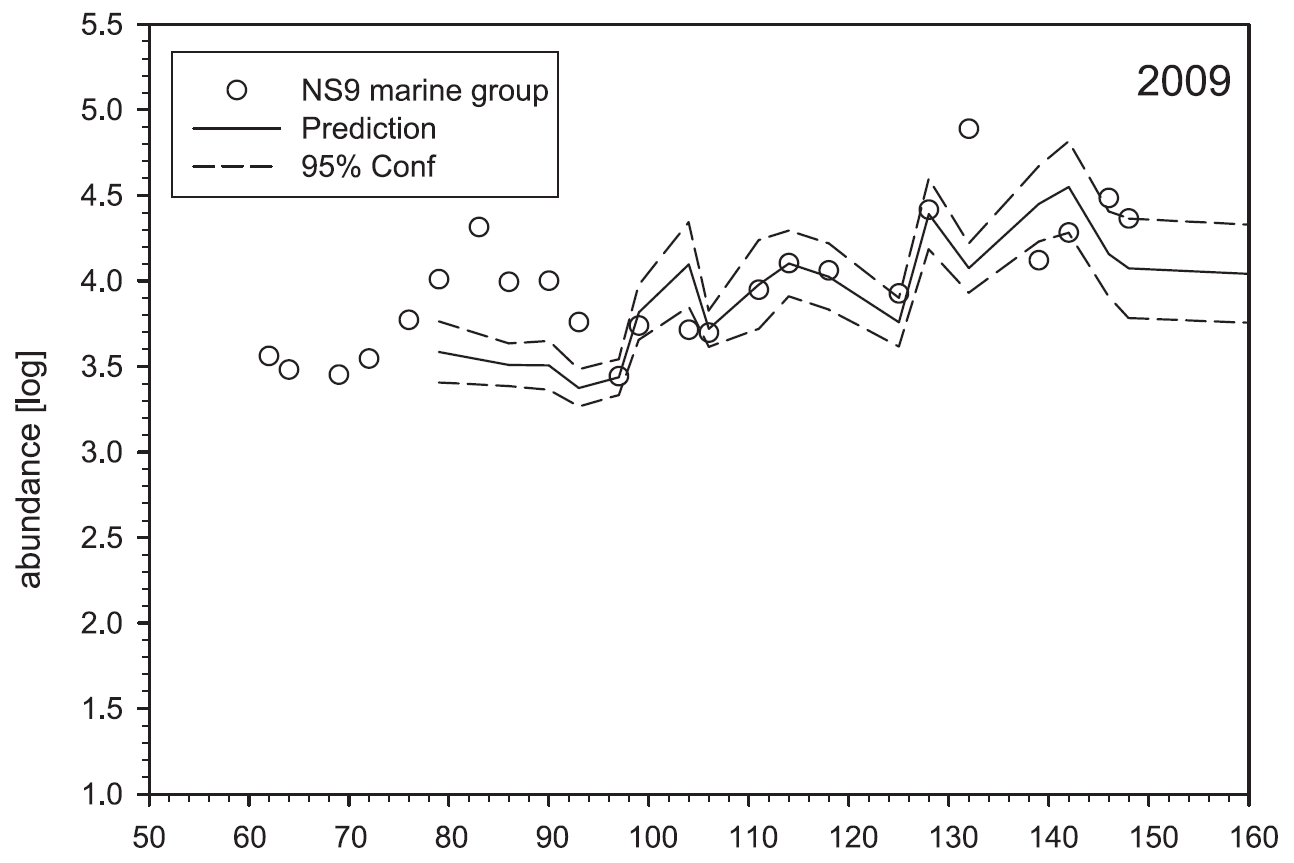


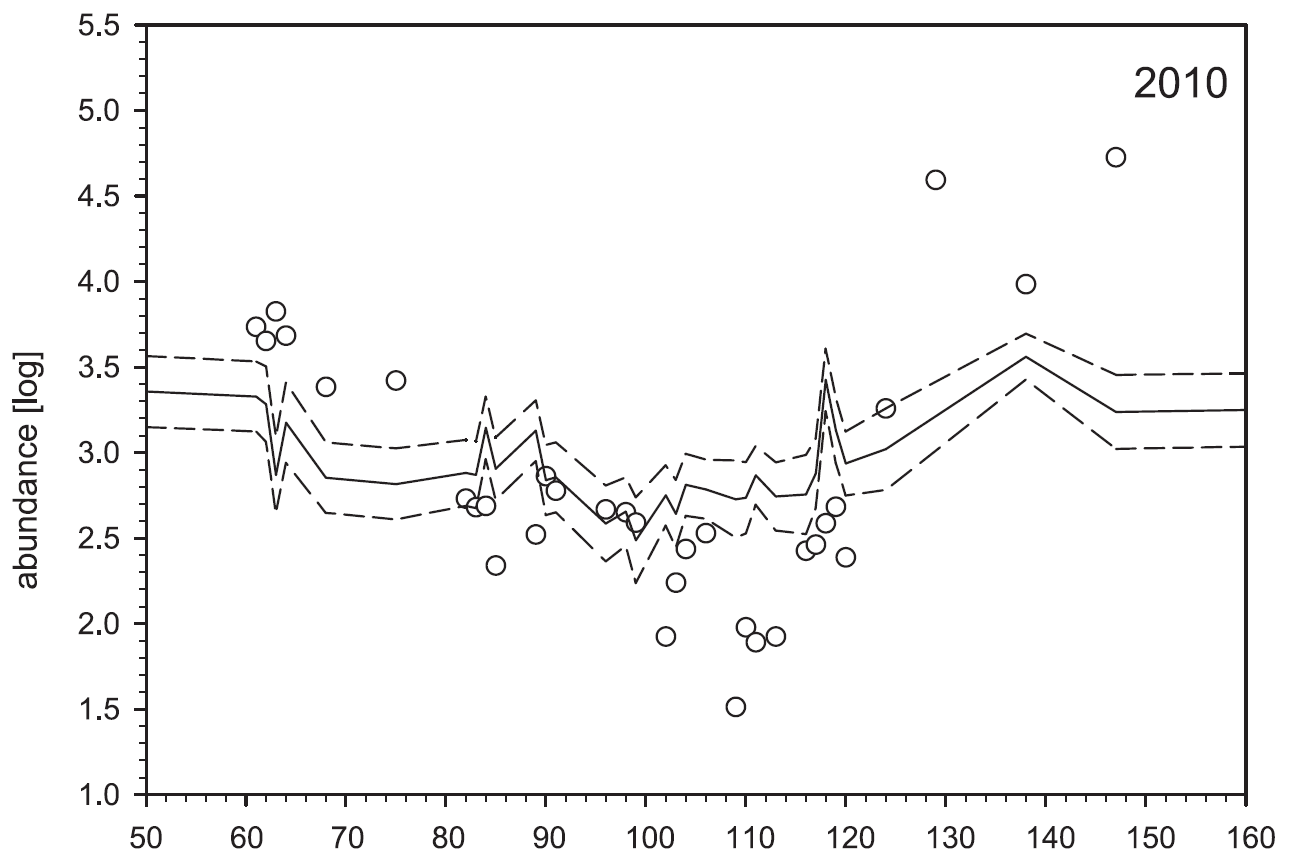


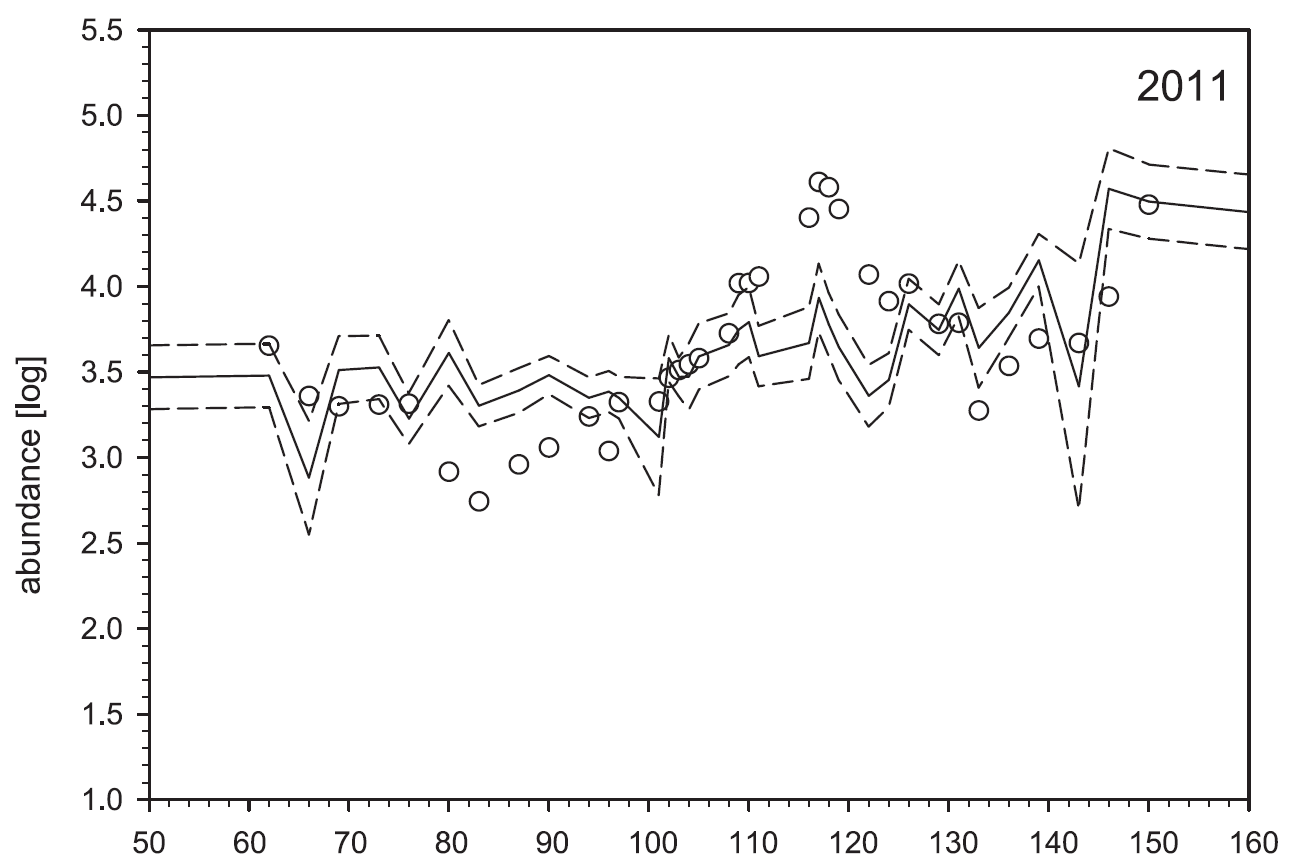


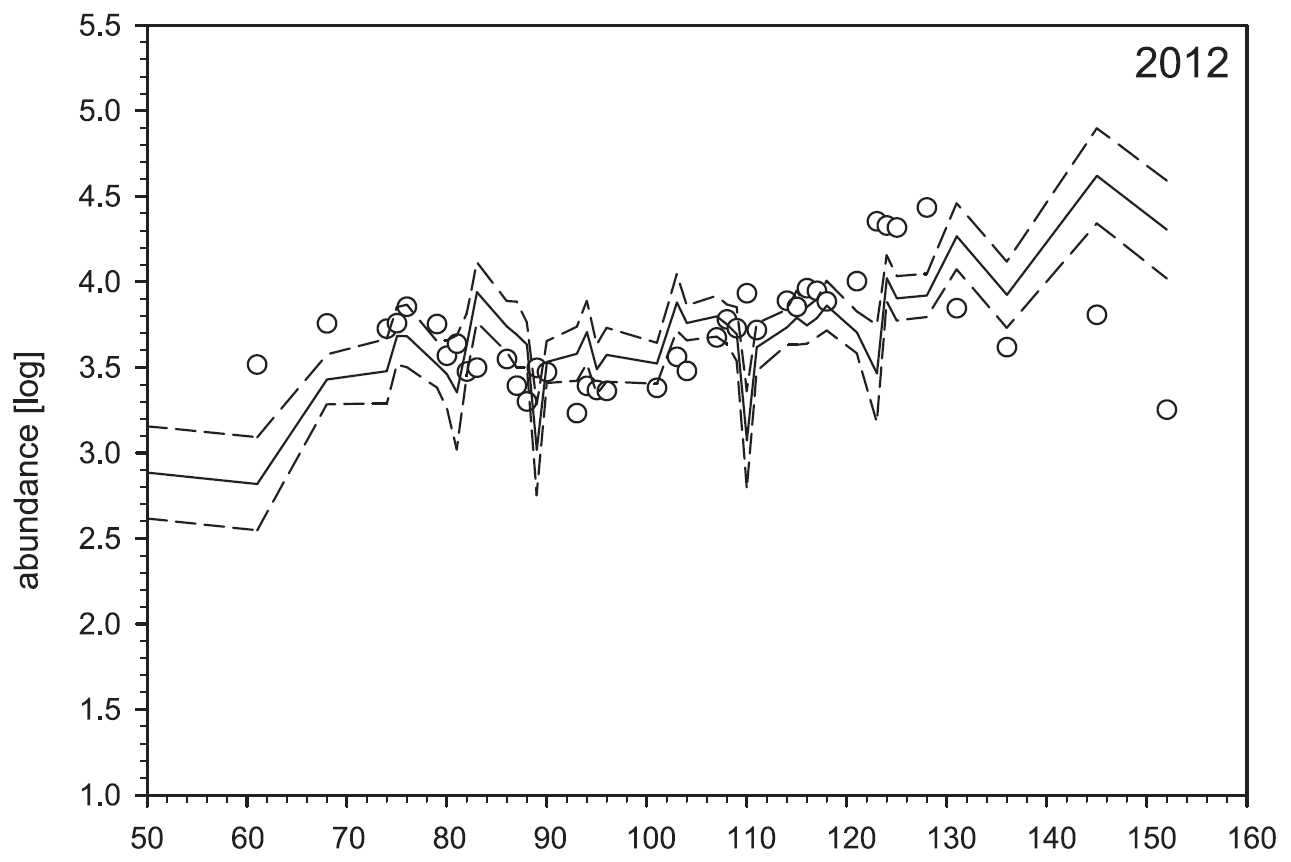


Julian day

**Linear regression analysis**

**- *Formosa* clade B (March-May 2009-2012) -**

independent variables:

Temperature, Salinity, Silicate, Phosphate, Nitrite, Nitrate, Ammonium, Greenalgae, Silicoflagellates, Coccolithophorids, Flagellates, Ciliates, *Mediopyxis, Chaetoceros debilis, Chaetoceros minimus, Rhizosolenia, Thalassosira, Dinophyceae, Phaeocystis, Chattonella*

The dependent variable *Formosa* clade B can be predicted from a linear combination of the independent variables:

Summary Table

| Step # | Variables Entered | R | RSqr | P |
| --- | --- | --- | --- | --- |
| 1 | Silicate | 0.398 | 0.159 | <0.001 |
| 2 | *Rhizosolenia styliformis* | 0.554 | 0.306 | <0.001 |
| 3 | *Chaet. debilis* | 0.596 | 0.355 | <0.001 |
| 4 | *Chattonella* | 0.64 | 0.409 | <0.001 |
| 5 | Ciliates | 0.668 | 0.447 | 0.009 |
| 6 | Salinity | 0.686 | 0.471 | <0.001 |
| 7 | Greenalgae | 0.706 | 0.498 | 0.004 |
| 8 | Temperature | 0.71 | 0.504 | 0.025 |


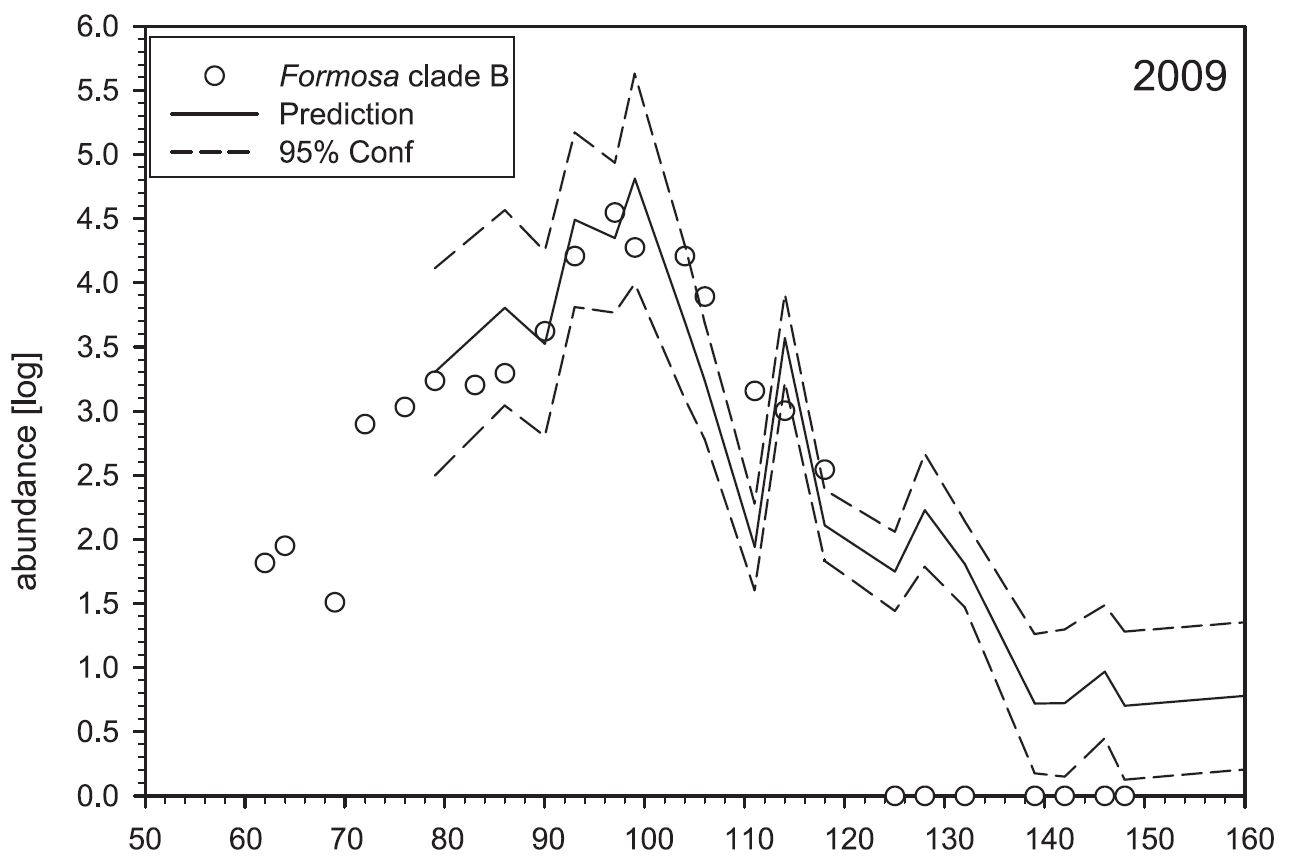


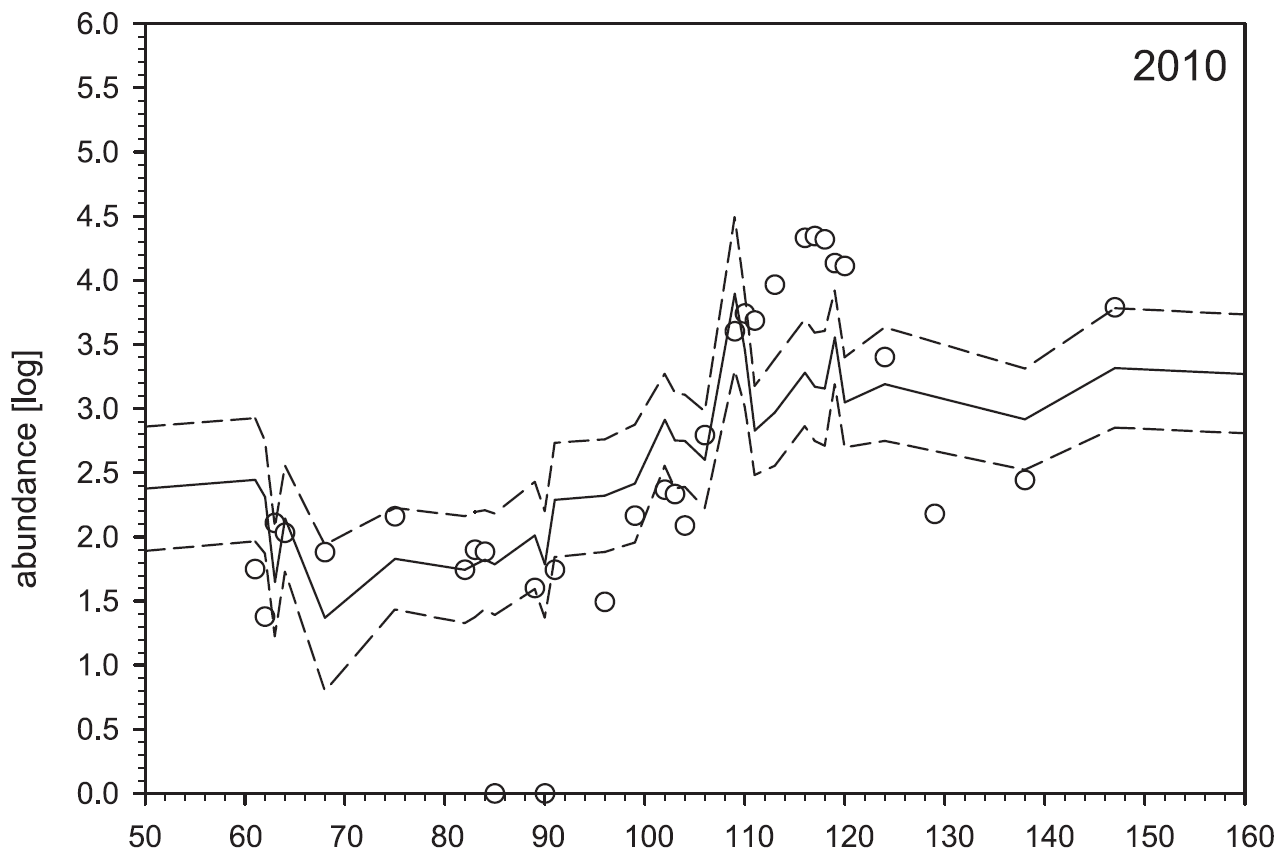


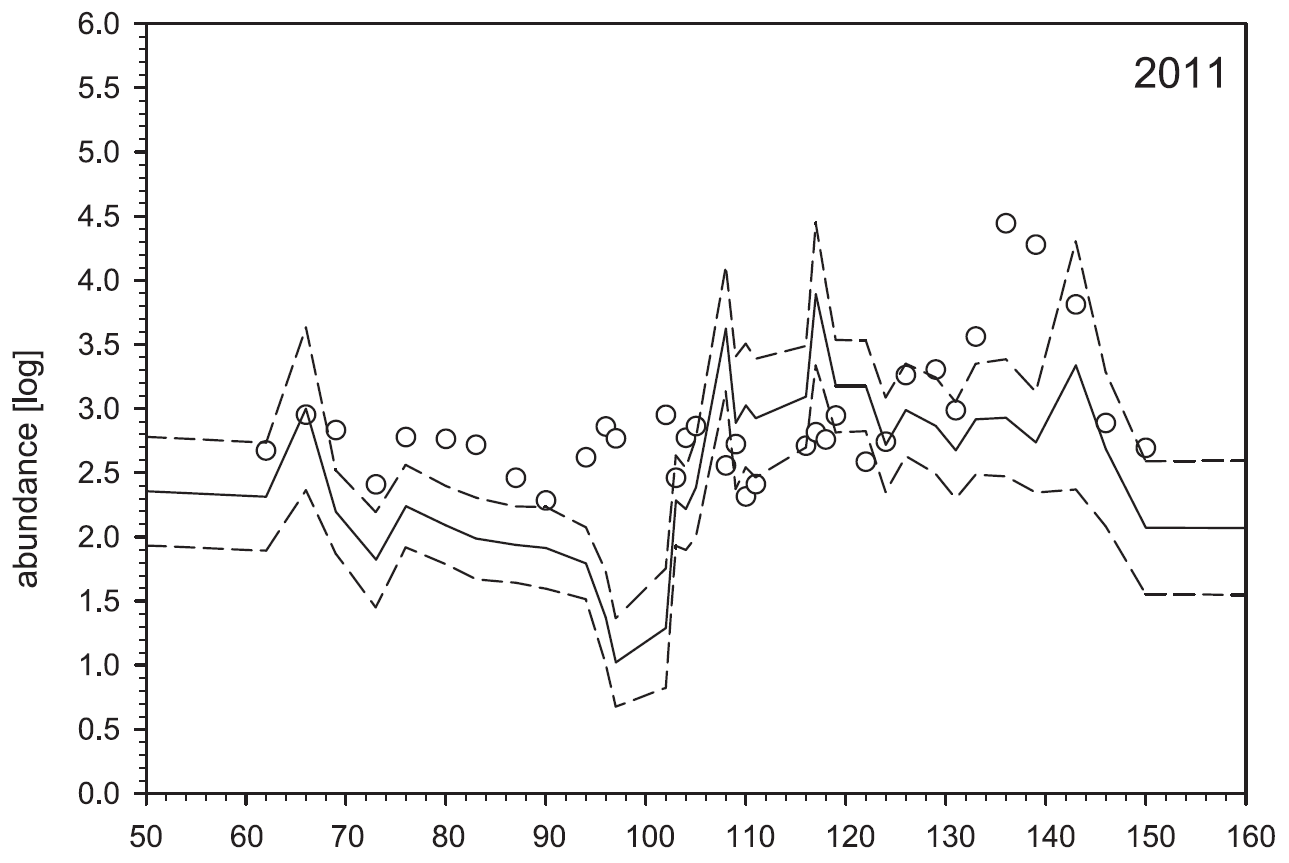


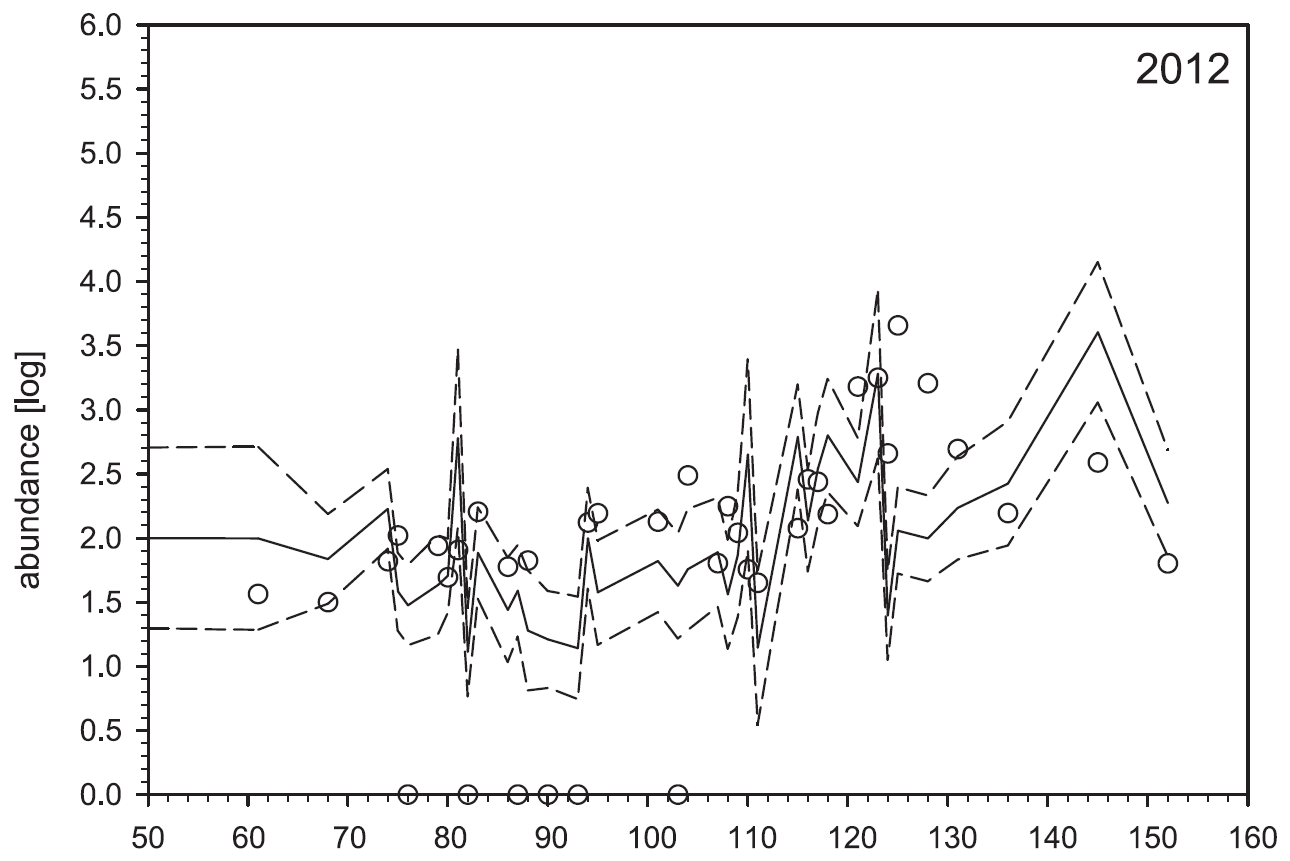


Julian day

**Linear regression analysis**

**- *Ulvibacter* (March-May 2009-2012) -**

independent variables:

Temperature, Salinity, Silicate, Phosphate, Nitrite, Nitrate, Ammonium, Greenalgae, Silicoflagellates, Coccolithophorids, Flagellates, Ciliates, *Mediopyxis, Chaetoceros debilis, Chaetoceros minimus, Rhizosolenia, Thalassosira, Dinophyceae, Phaeocystis, Chattonella*

The dependent variable *Ulvibacter* can be

predicted from a linear combination of the

independent variables:

Summary Table

| Step # | Variables Entered | R | RSqr | P |
| --- | --- | --- | --- | --- |
| 1 | Silicate | 0.803 | 0.645 | <0.001 |
| 2 | *Chaet. minimus* | 0.864 | 0.747 | <0.001 |
| 3 | *Mediopyxis helysia* | 0.874 | 0.764 | 0.001 |
| 4 | Silicoflagellates | 0.879 | 0.773 | 0.012 |
| 5 | Temperature | 0.884 | 0.782 | <0.001 |
| 6 | Nitrate | 0.894 | 0.798 | <0.001 |


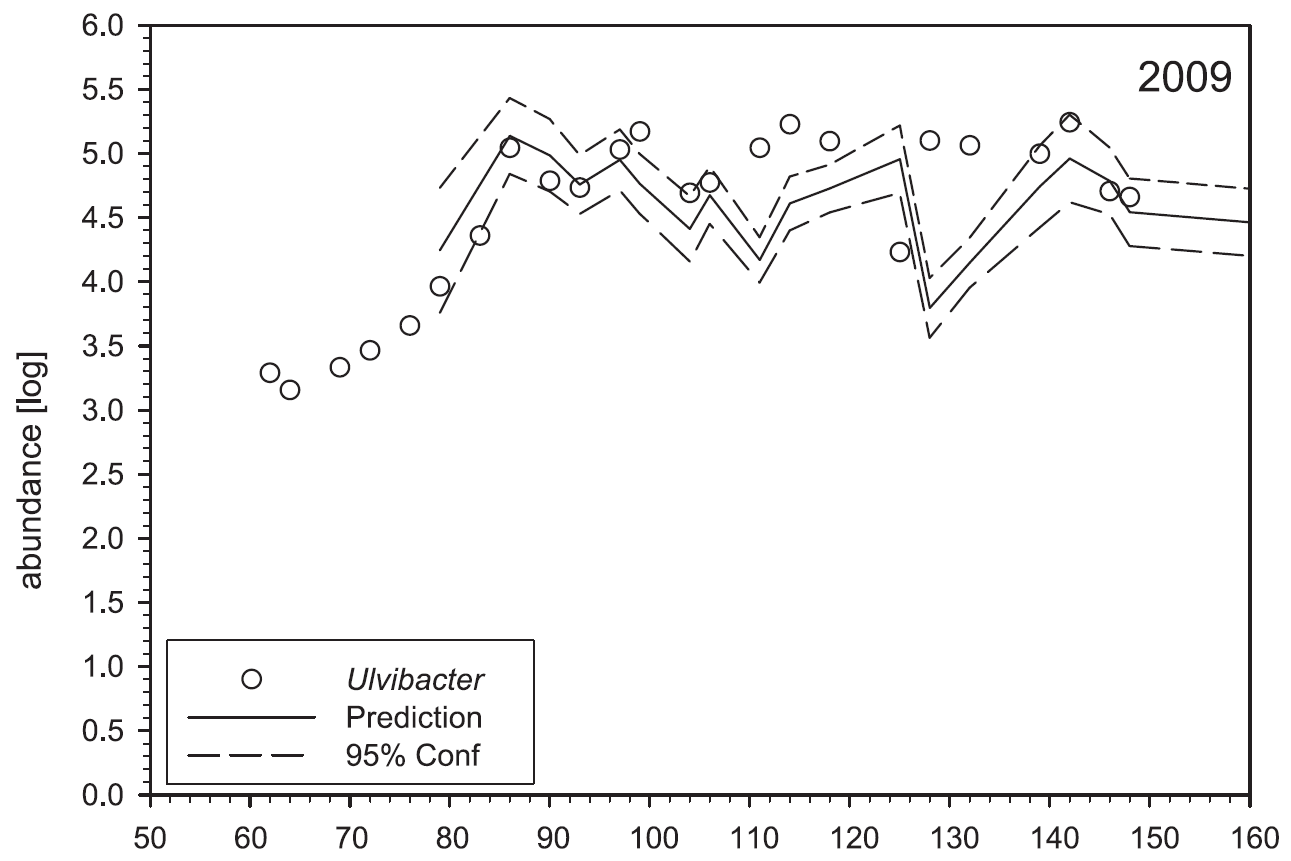


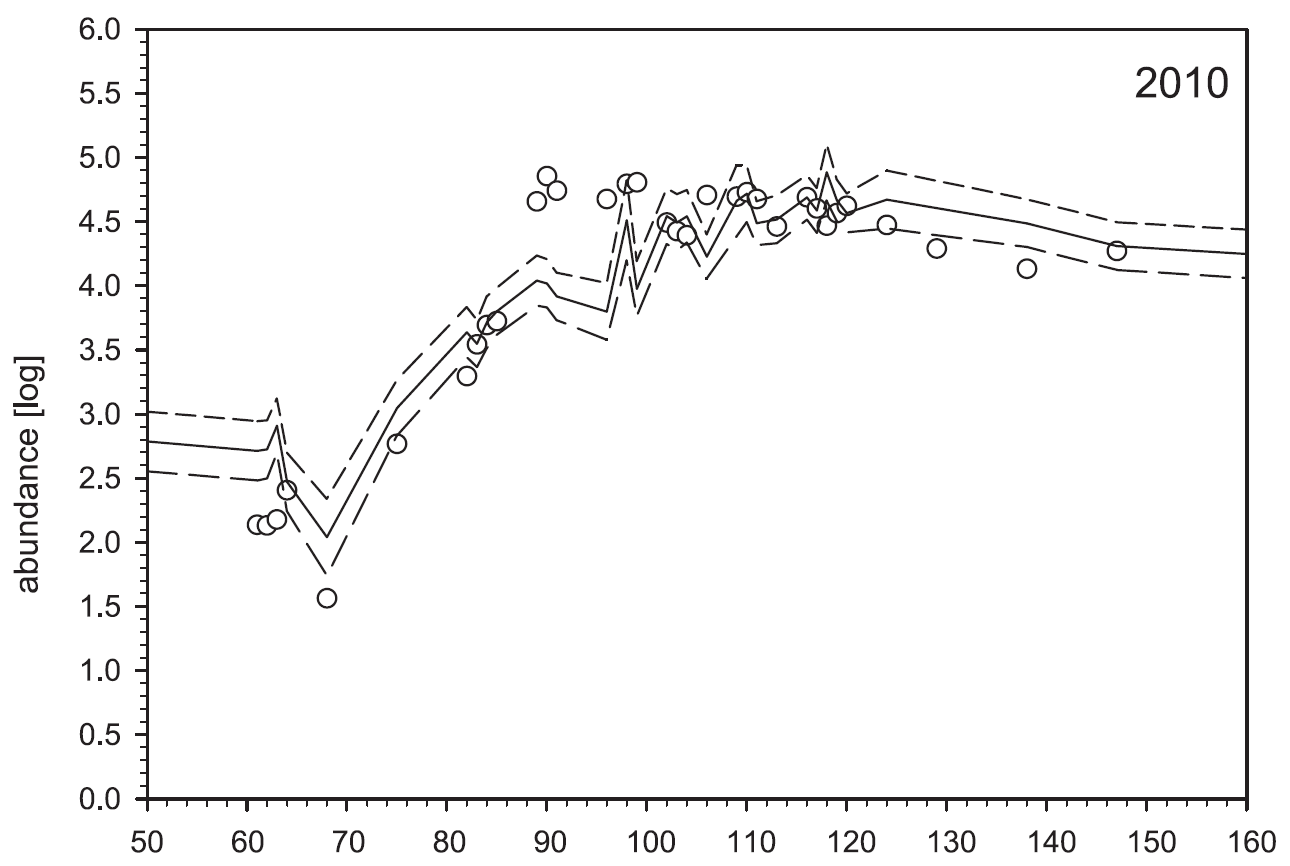


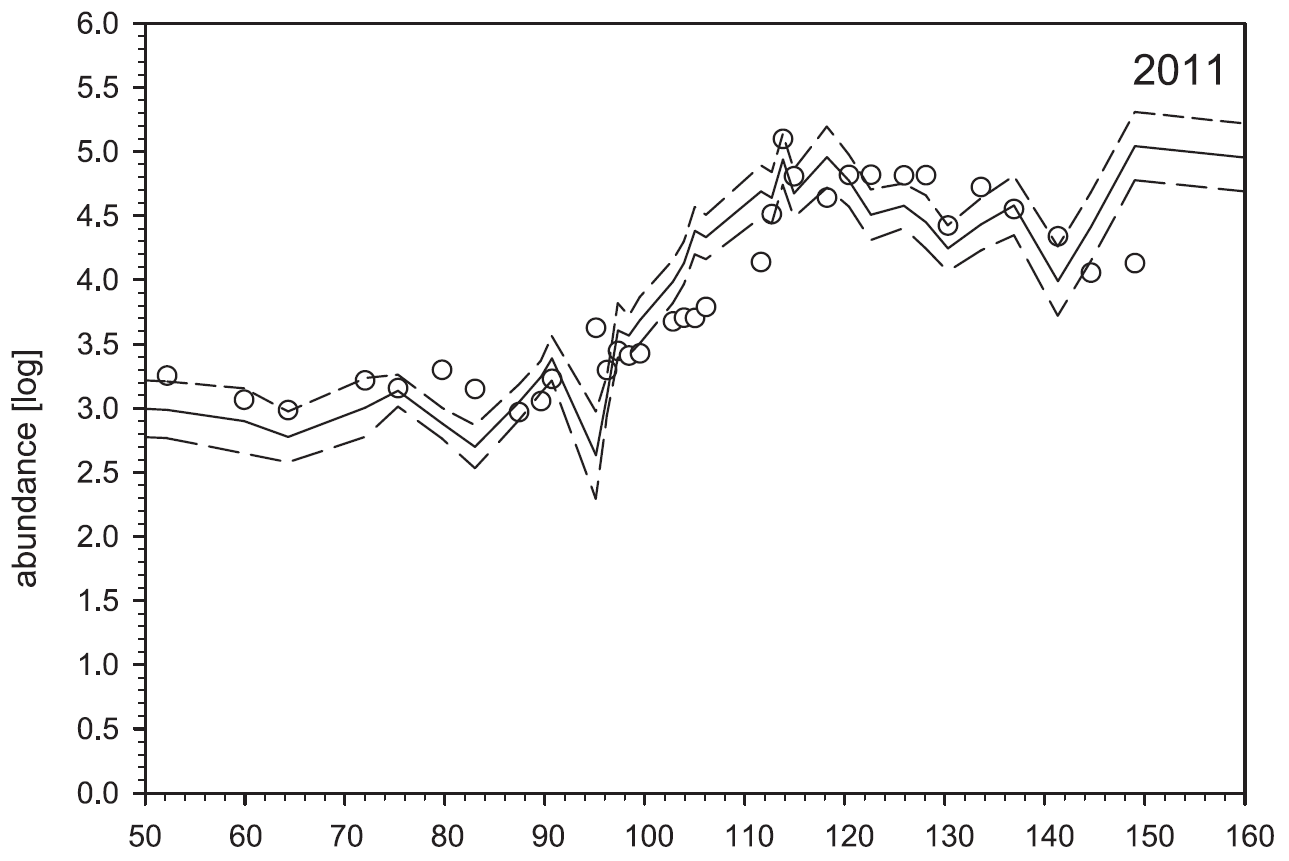


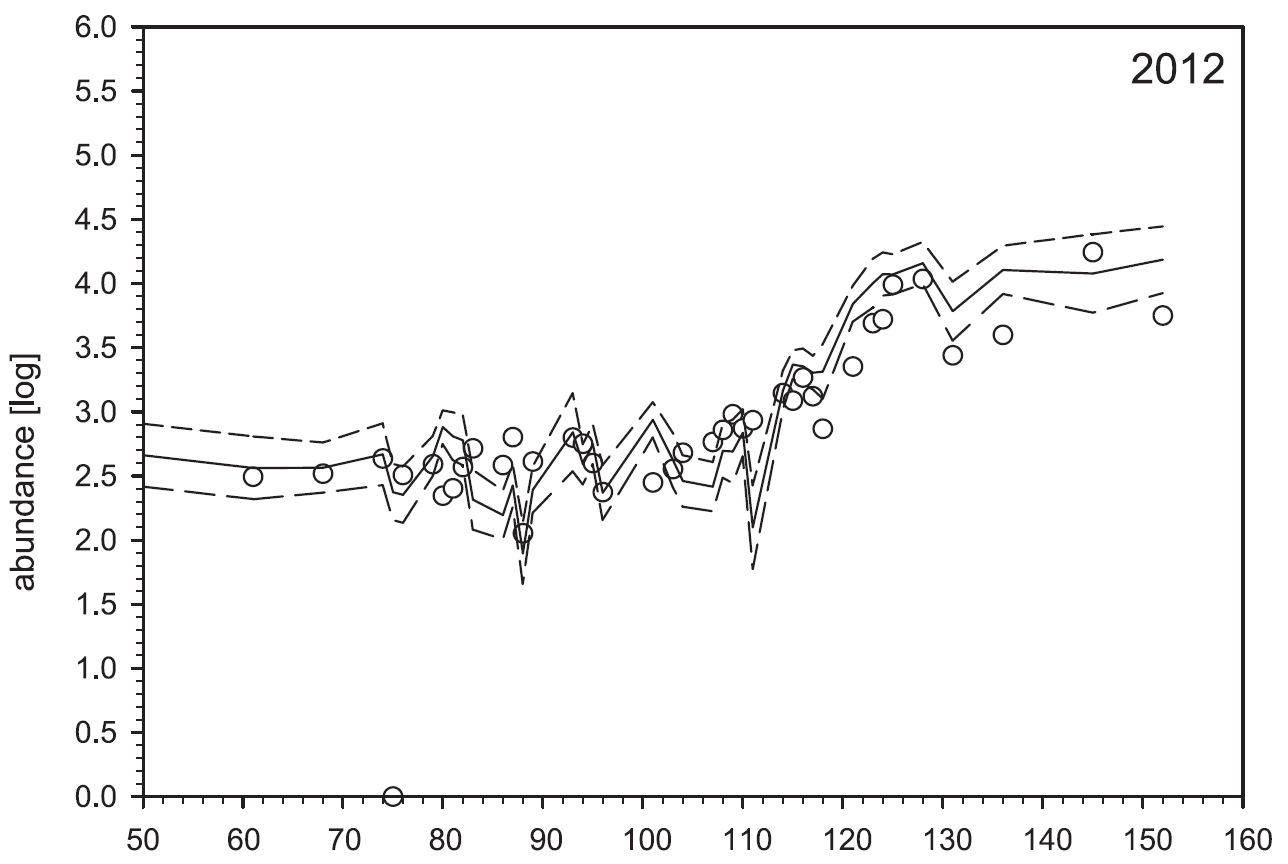


Julian day

**Linear regression analysis**

**- *Cryomorphaceae* (March-May 2009-2012) -**

independent variables:

Temperature, Salinity, Silicate, Phosphate, Nitrite, Nitrate, Ammonium, Greenalgae, Silicoflagellates, Coccolithophorids, Flagellates, Ciliates, *Mediopyxis, Chaetoceros debilis, Chaetoceros minimus, Rhizosolenia, Thalassosira, Dinophyceae, Phaeocystis, Chattonella*

The dependent variable *Cryomorphaceae* can be predicted from a linear combination of the independent variables:

Summary Table

| Step # | Variables Entered | R | RSqr | P |
| --- | --- | --- | --- | --- |
| 1 | Nitrate | 0.38 | 0.15 | 0.001 |
| 2 | *Chaet. debilis* | 0.46 | 0.212 | <0.001 |
| 3 | Nitrite | 0.501 | 0.251 | 0.005 |
| 4 | *Rhizosolenia styliformis* | 0.526 | 0.277 | <0.001 |
| 5 | *Phaeocystis* | 0.549 | 0.301 | 0.002 |
| 6 | Salinity | 0.595 | 0.355 | <0.001 |
| 7 | Silicoflagellates | 0.619 | 0.383 | 0.032 |
| 8 | *Chaet. minimus* | 0.635 | 0.403 | 0.047 |


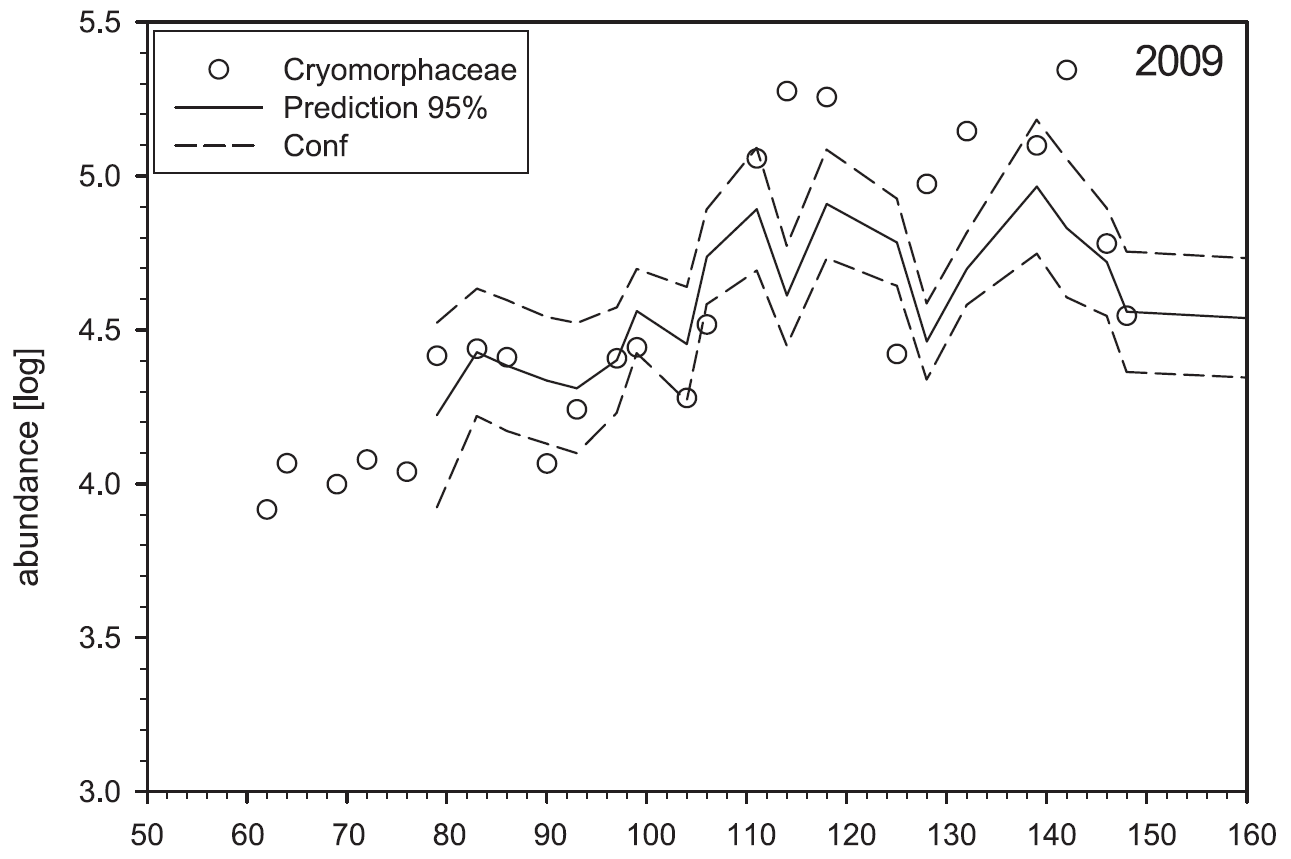


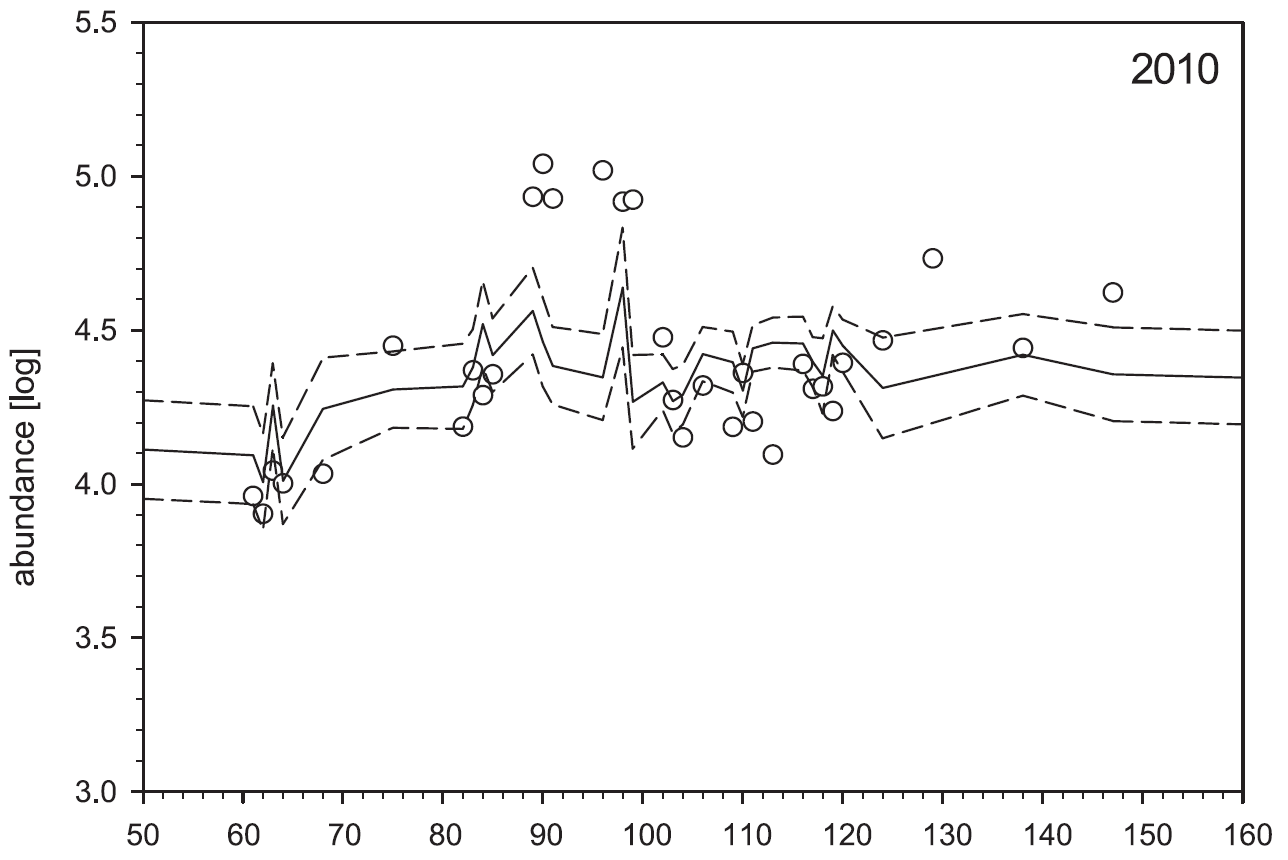


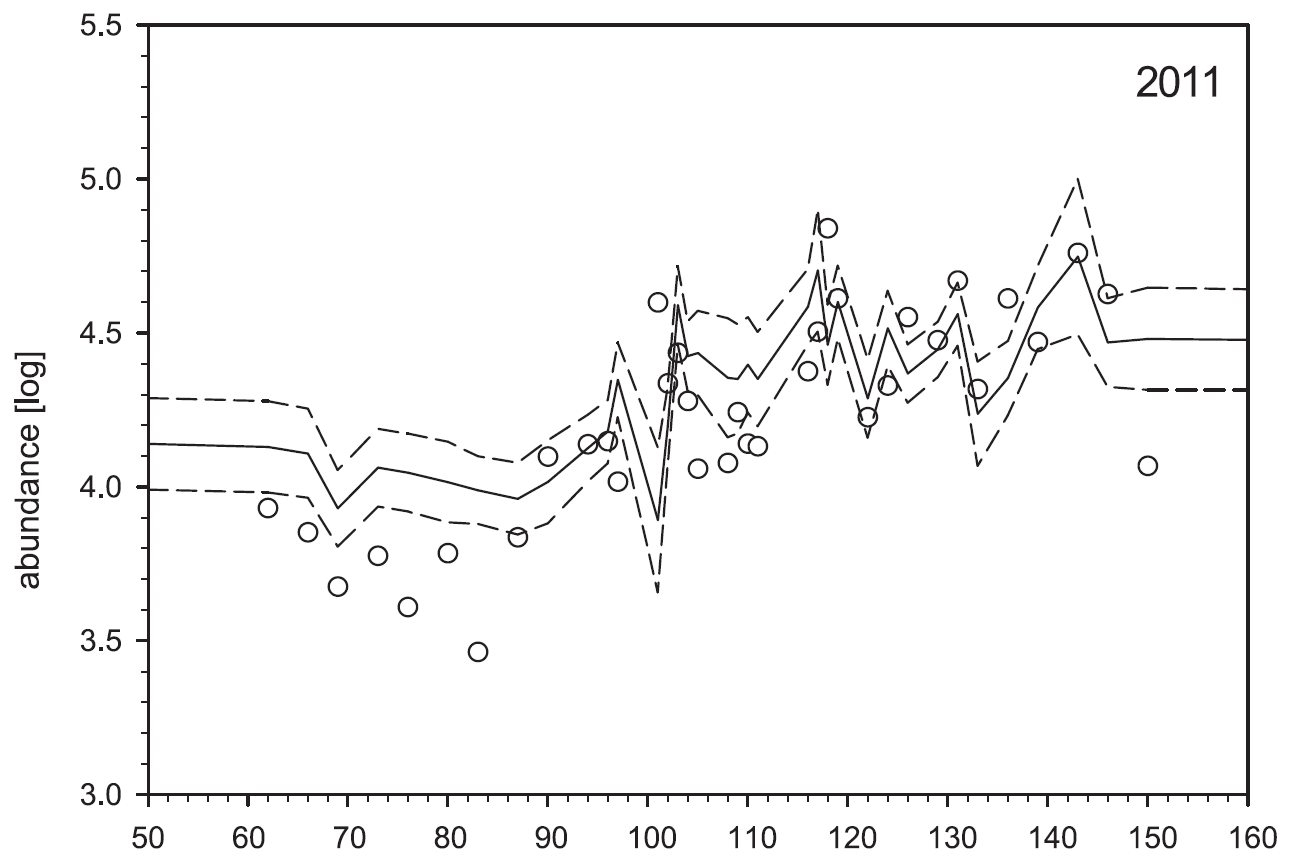


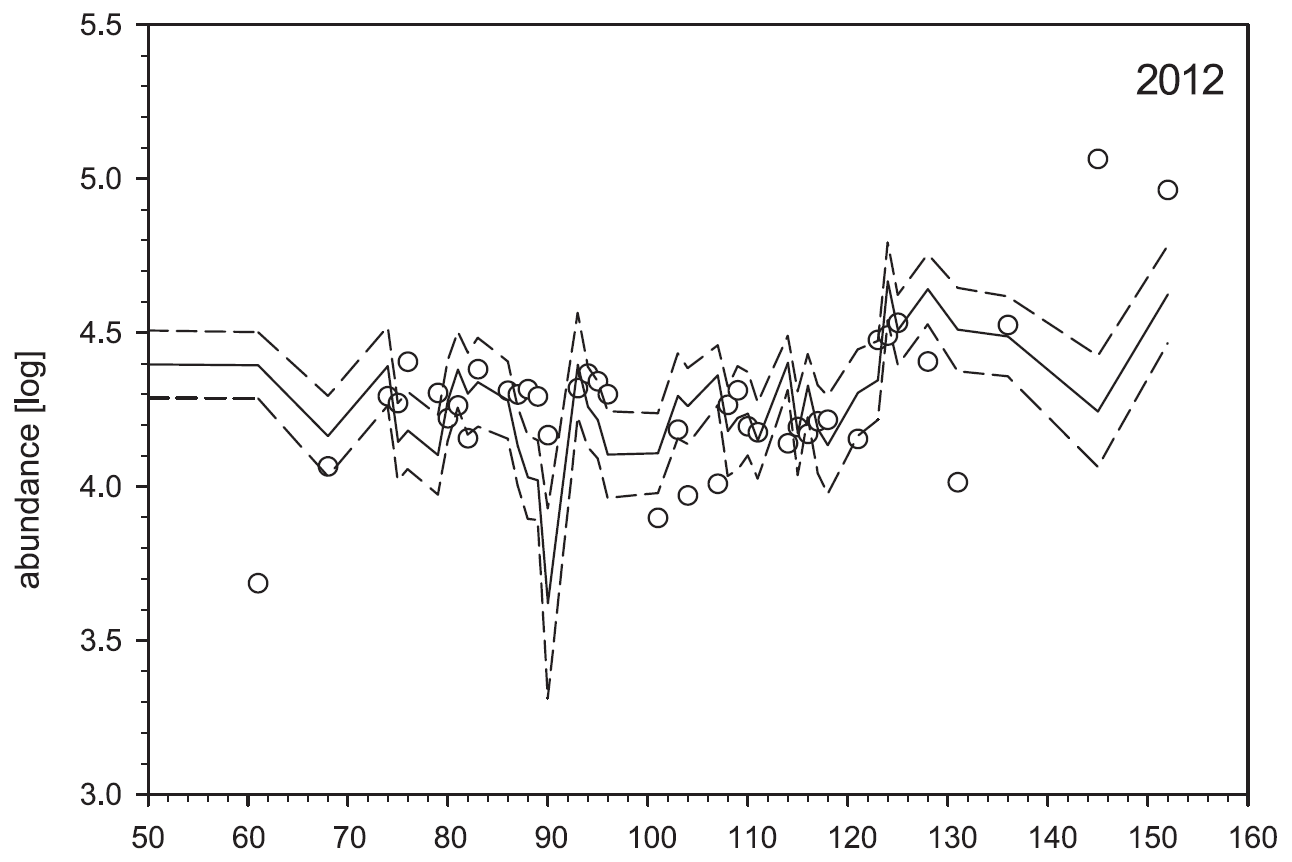


Julian day

**Linear regression analysis**

**- NS5 marine group (March-May 2009-2012) -**

independent variables:

Temperature, Salinity, Silicate, Phosphate, Nitrite, Nitrate, Ammonium, Greenalgae, Silicoflagellates, Coccolithophorids, Flagellates, Ciliates, *Mediopyxis, Chaetoceros debilis, Chaetoceros minimus, Rhizosolenia, Thalassosira, Dinophyceae, Phaeocystis, Chattonella*

The dependent variable NS5 marine group/VlS1 can be predicted from a linear combination of the independent variables:

Summary Table

| Step # | Variables Entered | R | RSqr | P |
| --- | --- | --- | --- | --- |
| 1 | *Chaet. minimus* | 0.564 | 0.318 | <0.001 |
| 2 | Silicate | 0.699 | 0.488 | <0.001 |
| 3 | Temperature | 0.732 | 0.536 | <0.001 |
| 4 | *Rhizosolenia styliformis* | 0.749 | 0.561 | <0.001 |
| 5 | Nitrate | 0.764 | 0.583 | 0.002 |
| 6 | Silicoflagellates | 0.774 | 0.599 | 0.036 |


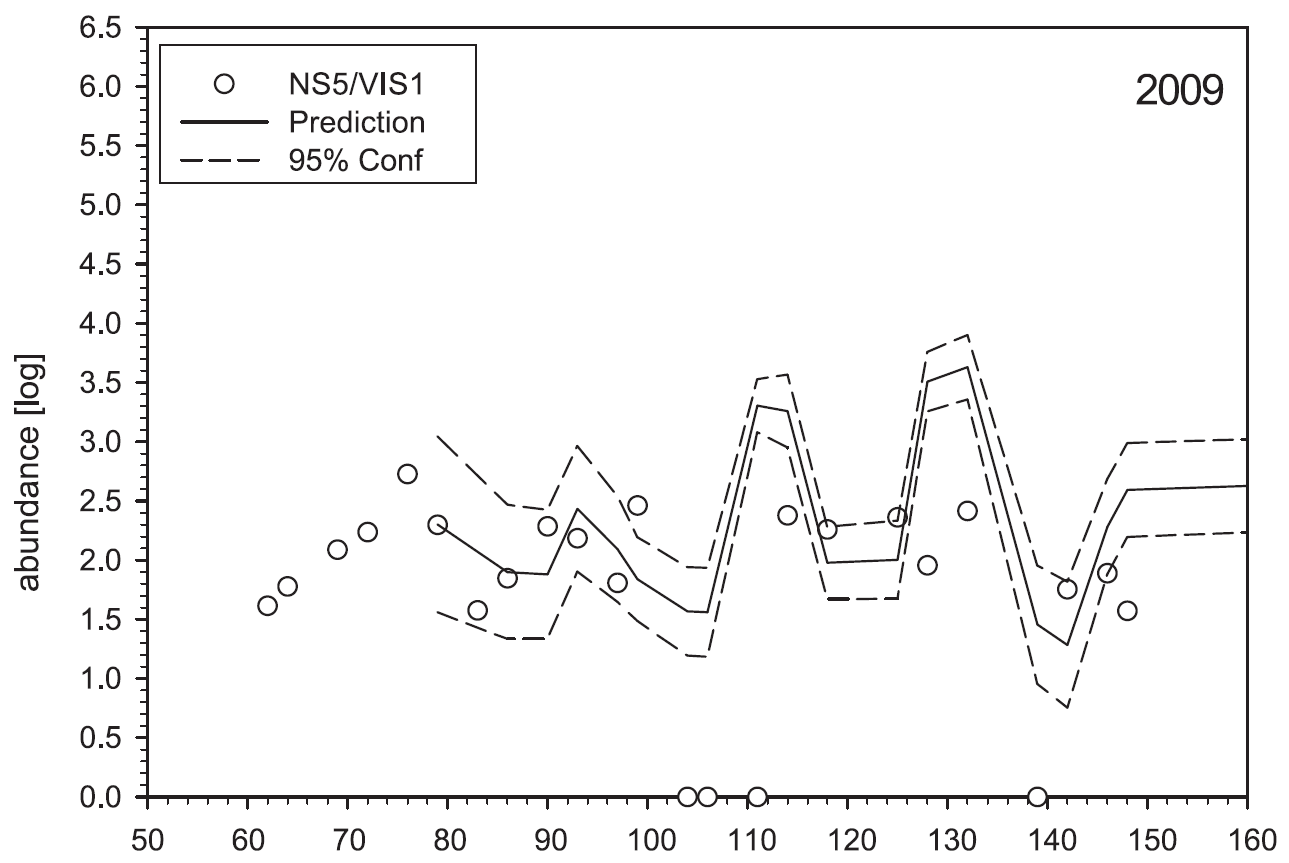


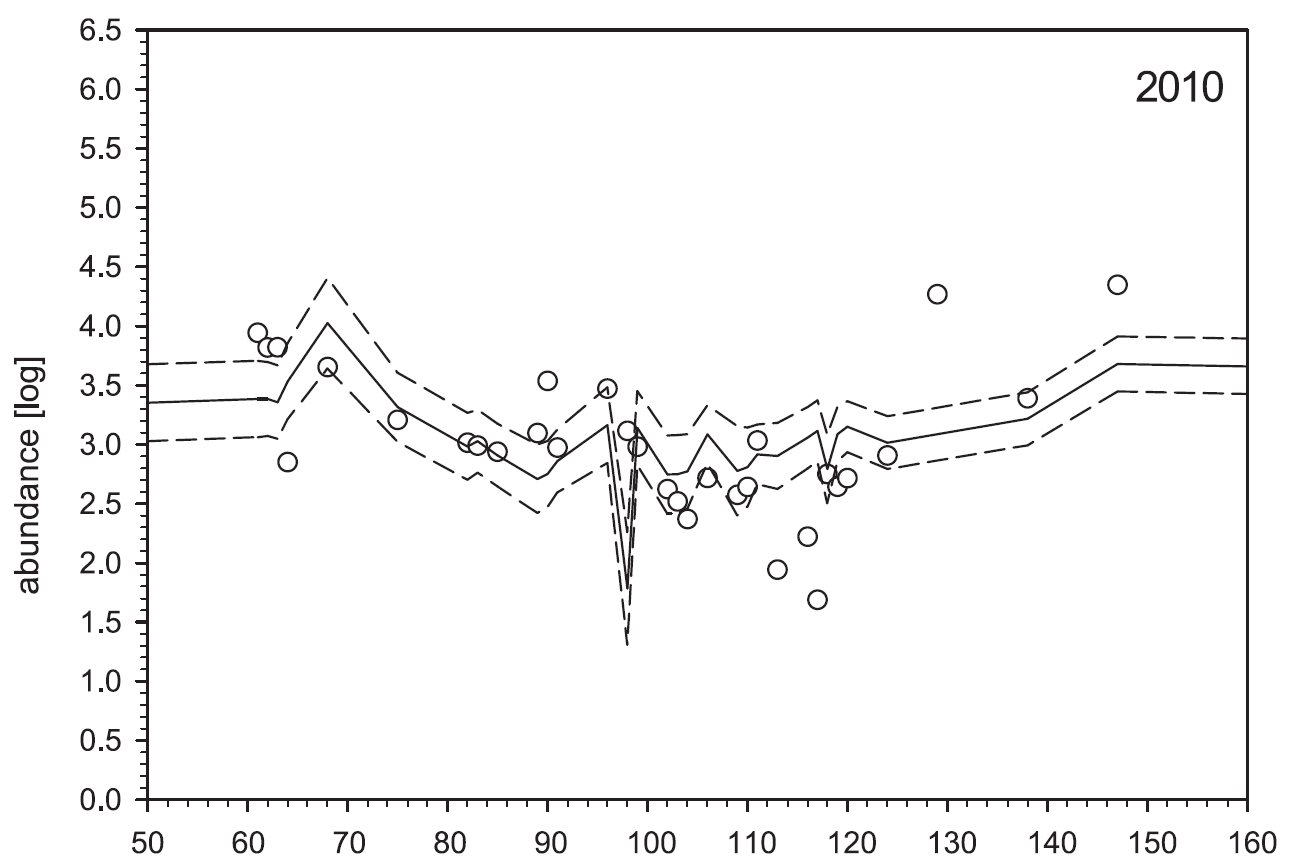


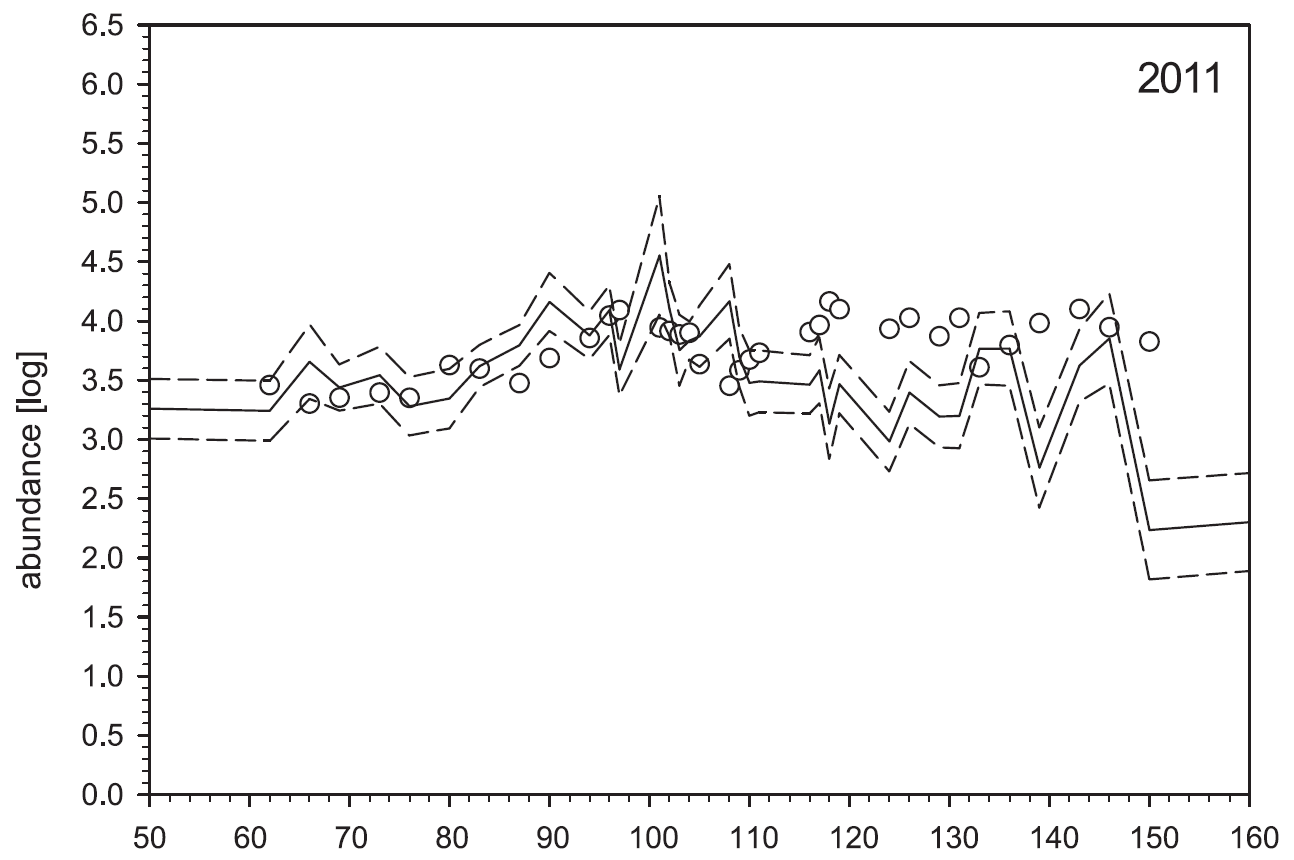


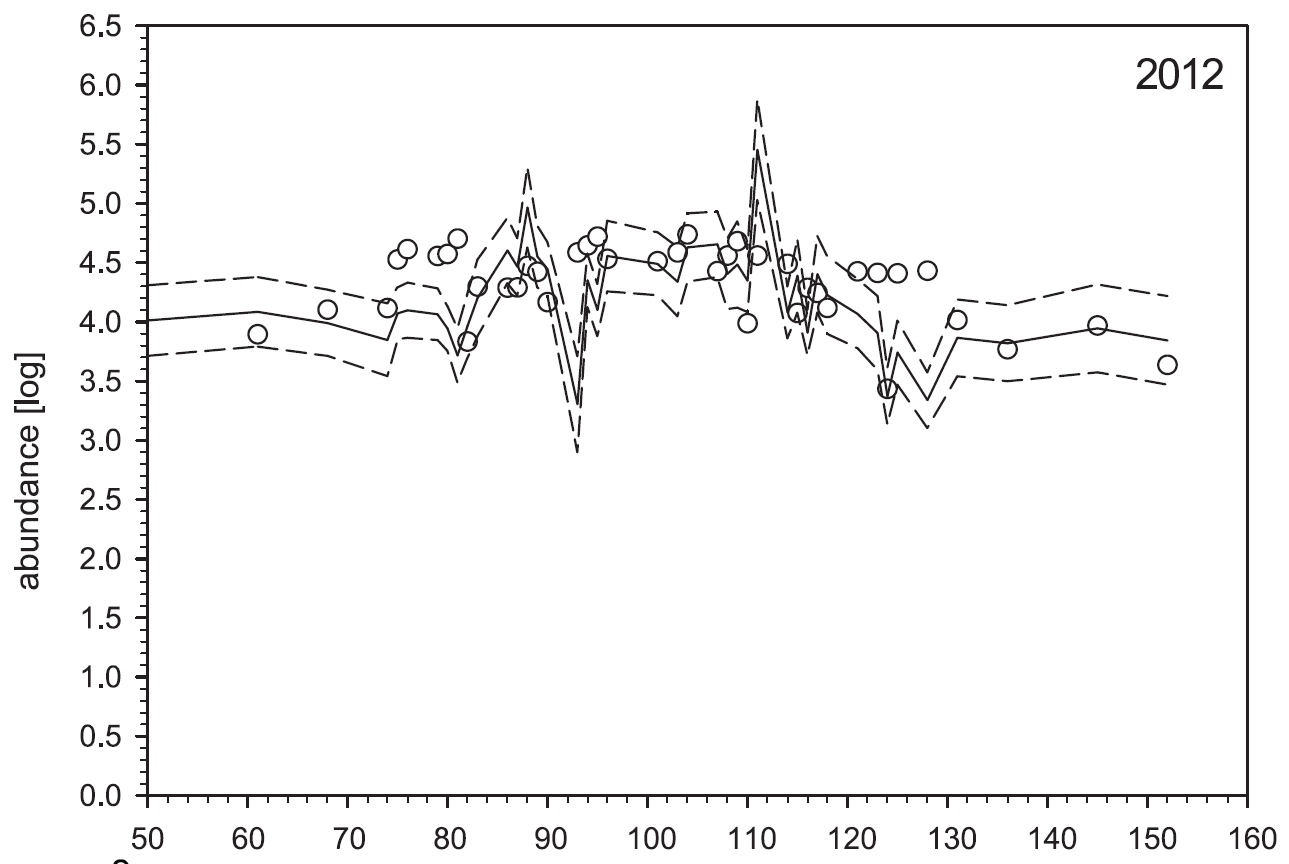


Julian day

**Linear regression analysis**

**- NS3 marine group (March-May 2009-2012) -**

independent variables:

Temperature, Salinity, Silicate, Phosphate, Nitrite, Nitrate, Ammonium, Greenalgae, Silicoflagellates, Coccolithophorids, Flagellates, Ciliates, *Mediopyxis, Chaetoceros debilis, Chaetoceros minimus, Rhizosolenia, Thalassosira, Dinophyceae, Phaeocystis, Chattonella*

The dependent variable NS3 marine group

can be predicted from a linear combination

of the independent variables:

Summary Table

| Step # | Variables Entered | R | RSqr | P |
| --- | --- | --- | --- | --- |
| 1 | Salinity | 0.372 | 0.138 | <0.001 |
| 2 | *Phaeocystis* | 0.513 | 0.263 | <0.001 |
| 3 | Nitrate | 0.57 | 0.33 | <0.001 |
| 4 | *Chattonella* | 0.622 | 0.387 | 0.001 |
| 5 | *Thalassiosira nordenskioeldii* | 0.644 | 0.415 | 0.001 |
| 6 | *Chaet. debilis* | 0.689 | 0.474 | <0.001 |
| 7 | *Rhizosolenia styliformis* | 0.702 | 0.493 | 0.005 |
| 8 | *Chaet. minimus* | 0.716 | 0.51 | 0.03 |


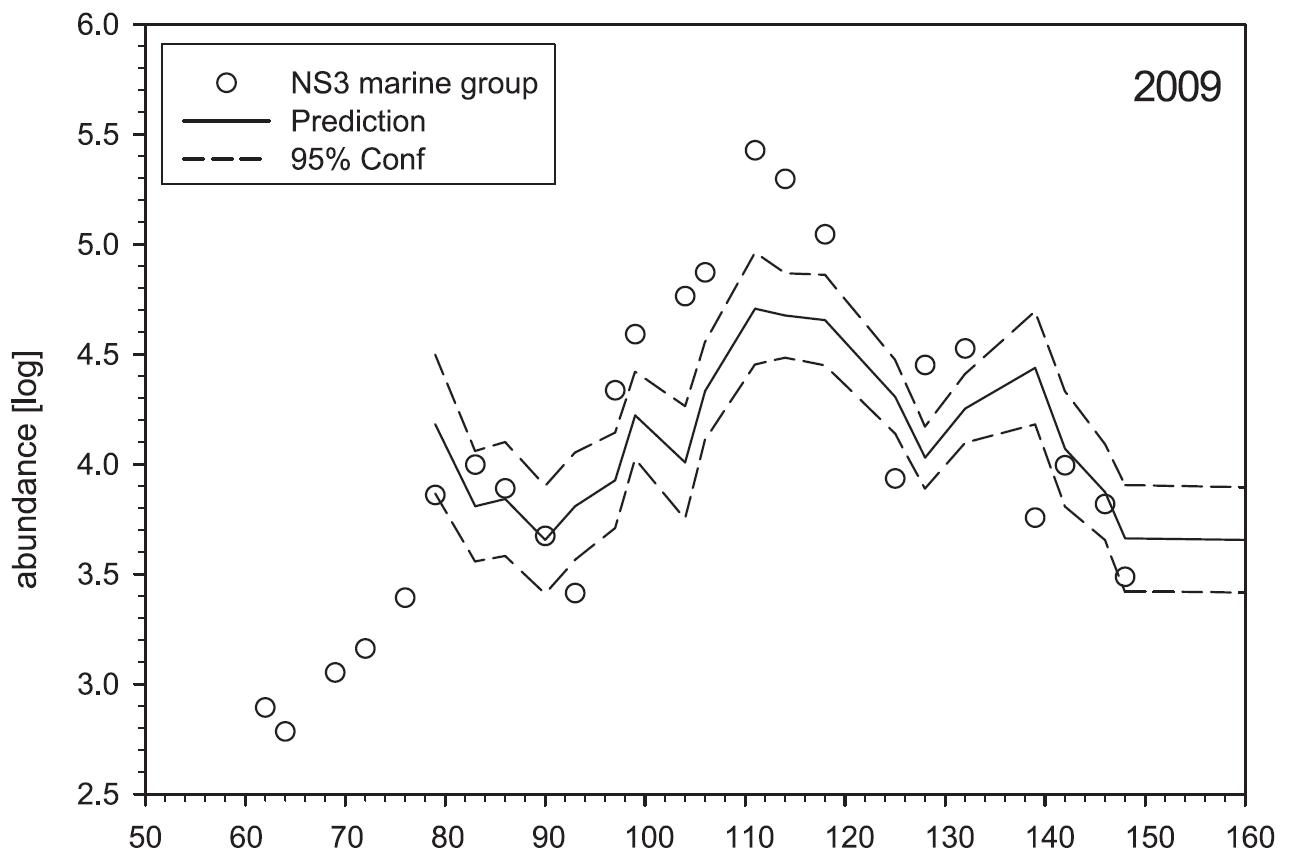


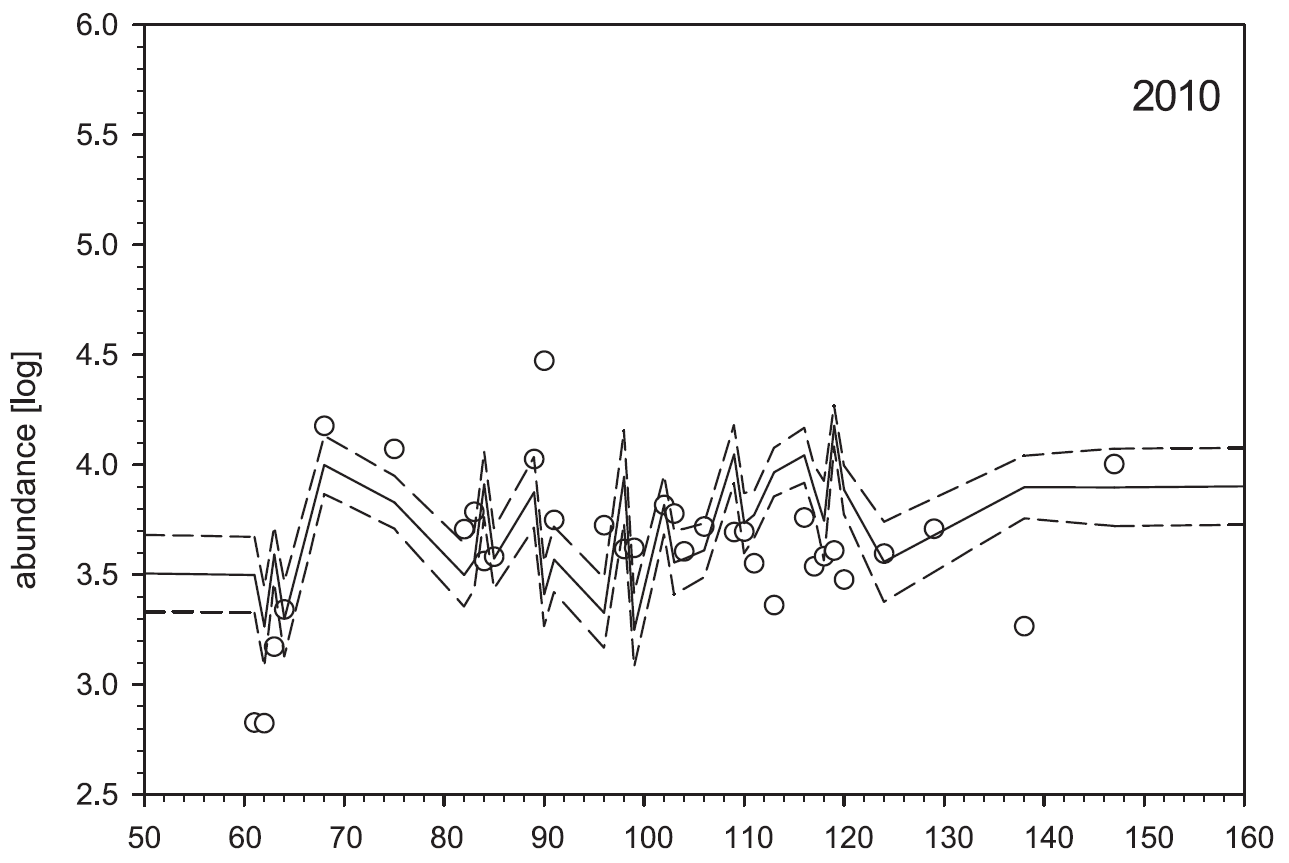


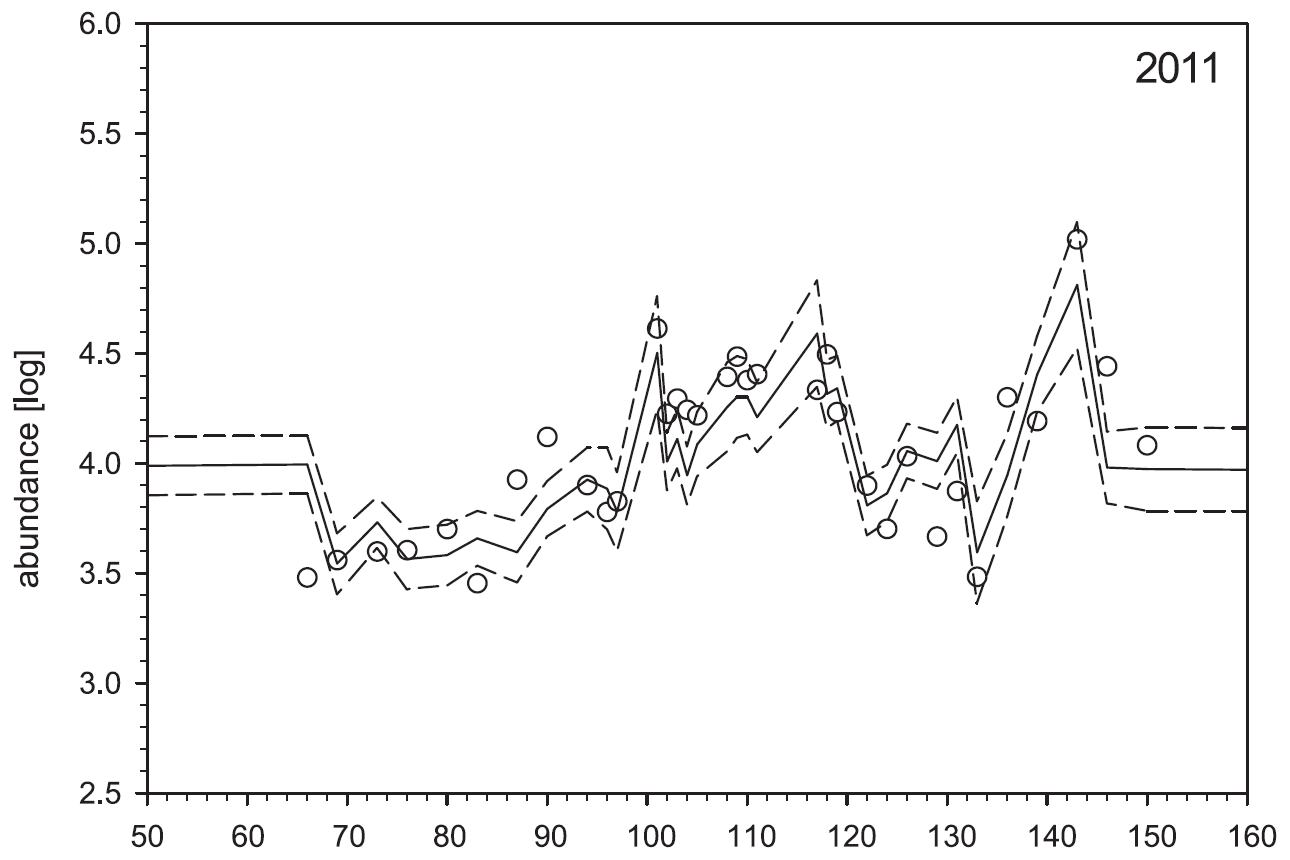


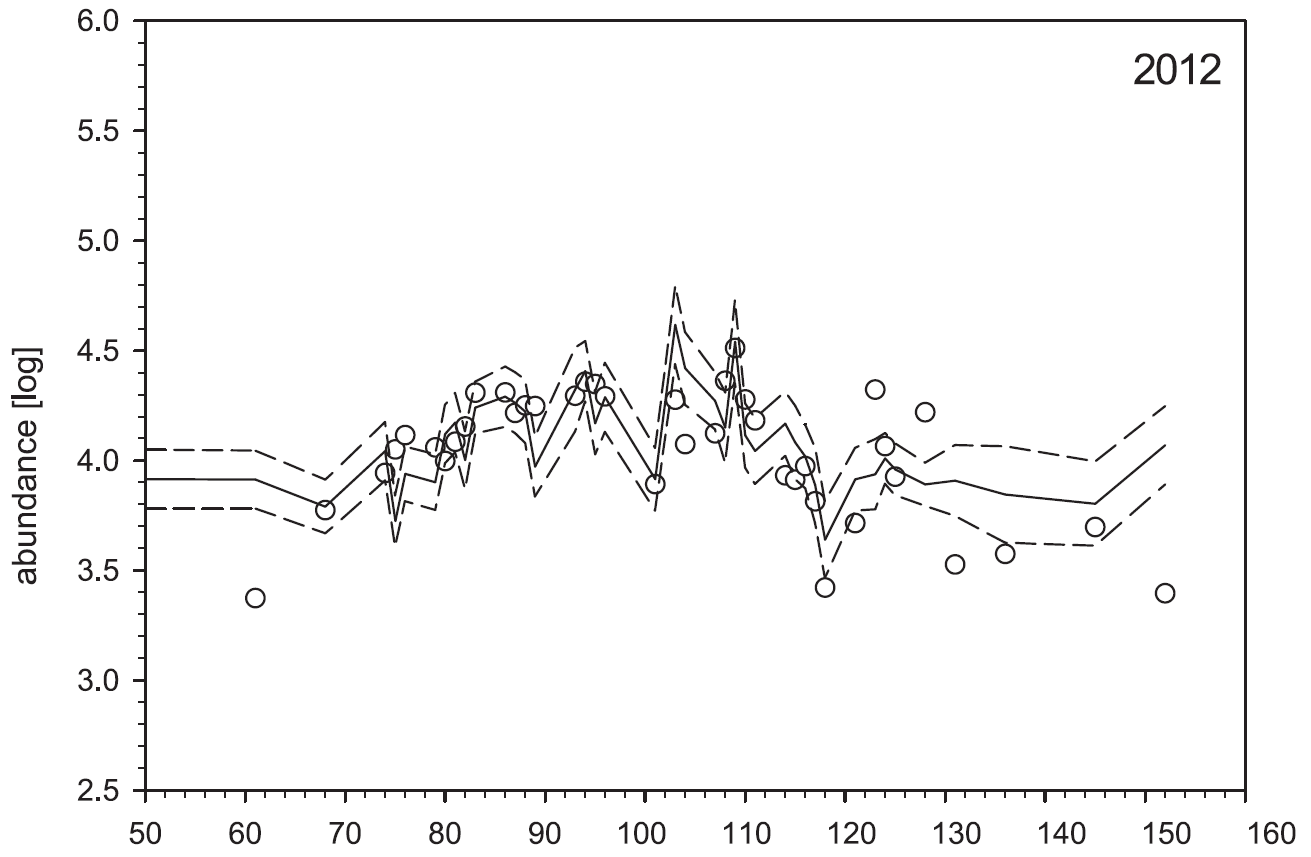


Julian day

**Linear regression analysis**

**- *Cytophagia* (March-May 2009-2012) -**

independent variables:

Temperature, Salinity, Silicate, Phosphate, Nitrite, Nitrate, Ammonium, Greenalgae, Silicoflagellates, Coccolithophorids, Flagellates, Ciliates, *Mediopyxis, Chaetoceros debilis, Chaetoceros minimus, Rhizosolenia, Thalassosira, Dinophyceae, Phaeocystis, Chattonella*

The dependent variable *Cytophagia* can be predicted from a linear combination of the independent variables:

Summary Table

| Step # | Variables Entered | R | RSqr | P |
| --- | --- | --- | --- | --- |
| 1 | Temperature | 0.488 | 0.239 | <0.001 |
| 2 | *Phaeocystis* | 0.542 | 0.293 | <0.001 |
| 3 | Flagellates | 0.588 | 0.345 | <0.001 |
| 4 | Ciliates | 0.627 | 0.393 | 0.01 |
| 5 | *Chaet. debilis* | 0.644 | 0.415 | <0.001 |
| 6 | *Mediopyxis helysia* | 0.671 | 0.451 | <0.001 |
| 7 | *Chaet. minimus* | 0.702 | 0.493 | 0.003 |


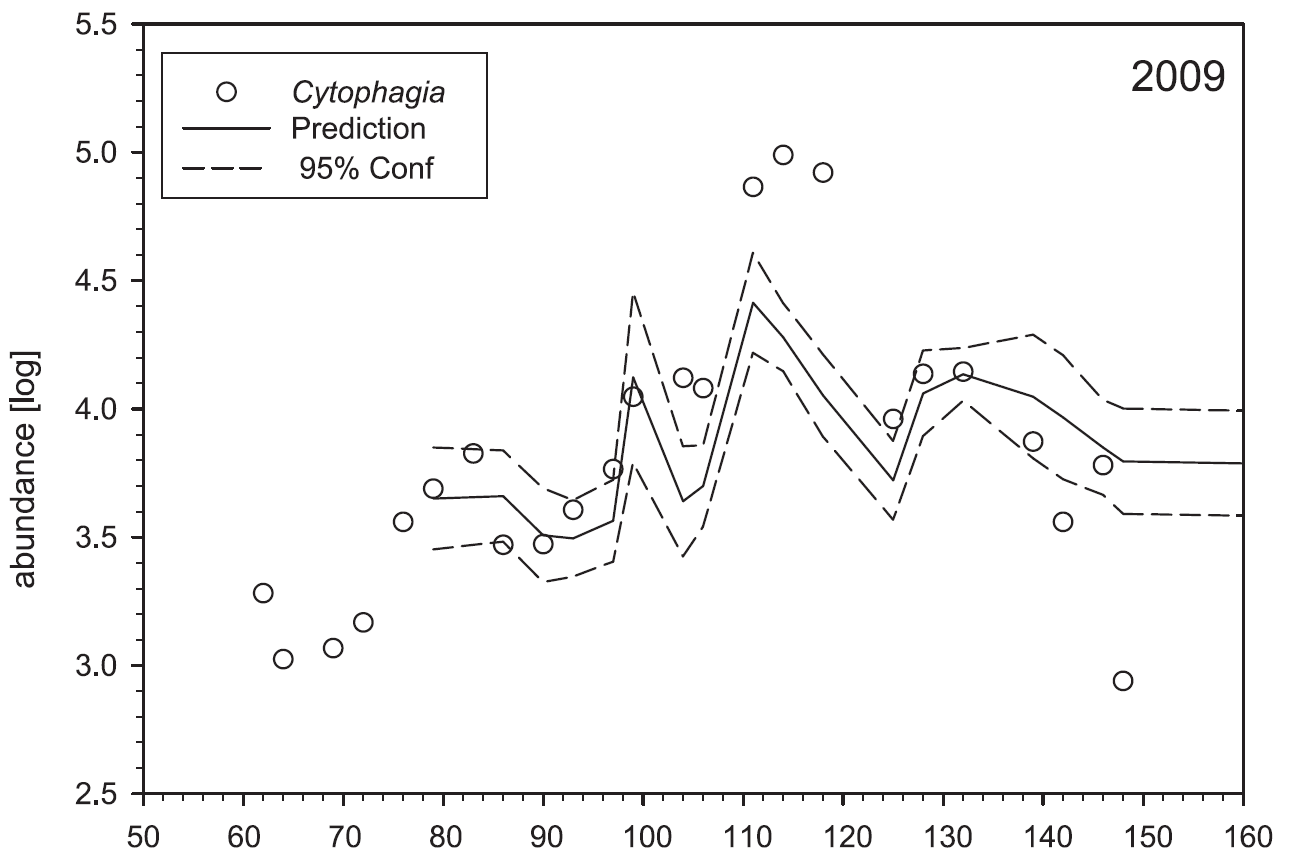


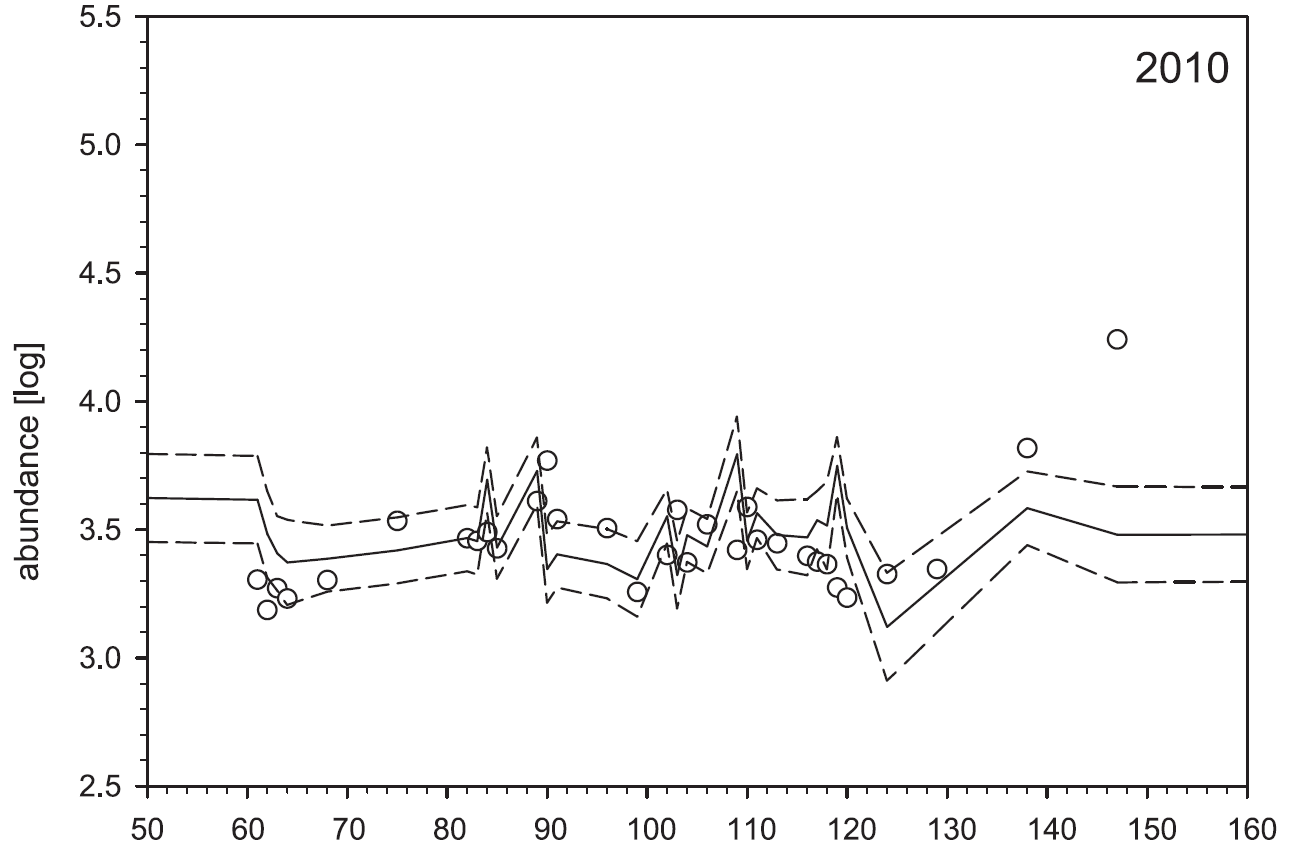


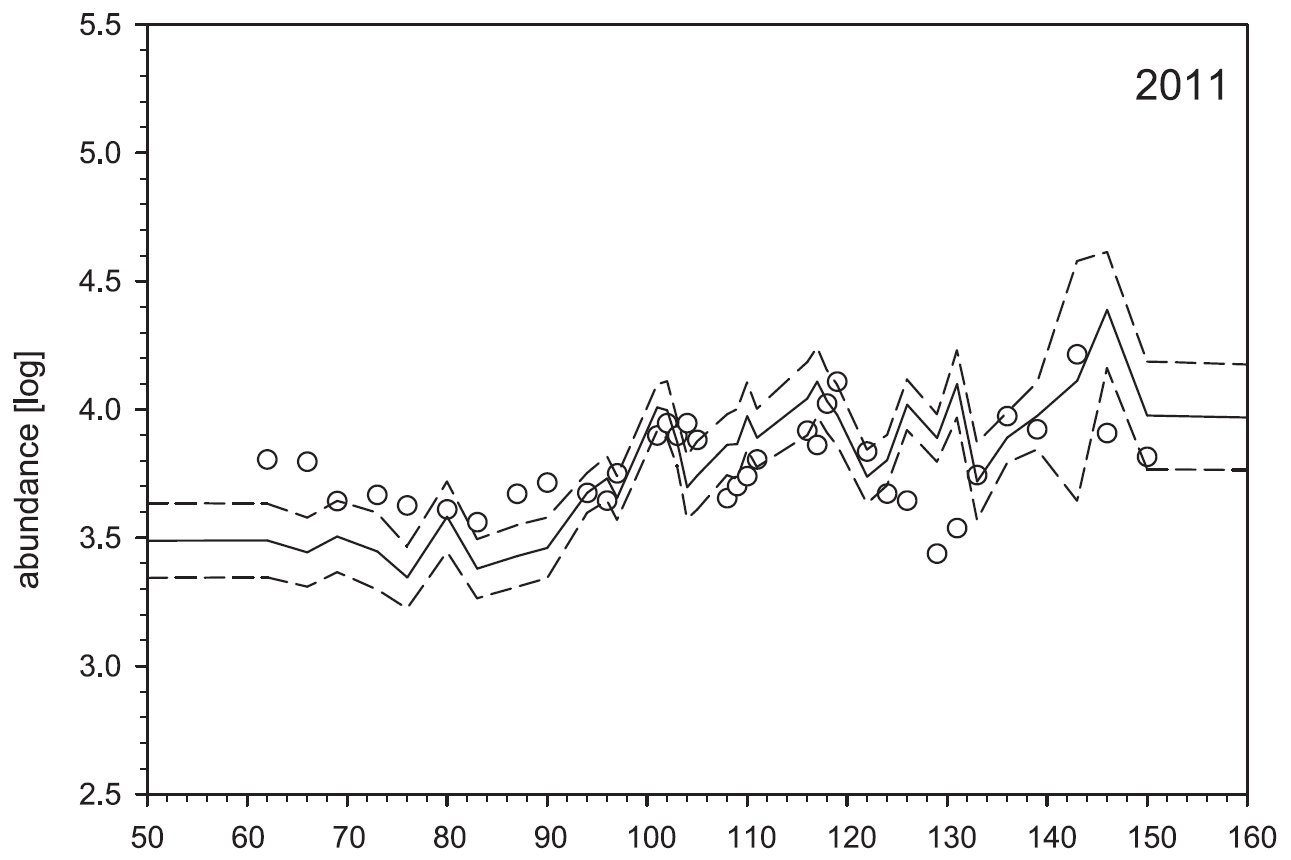


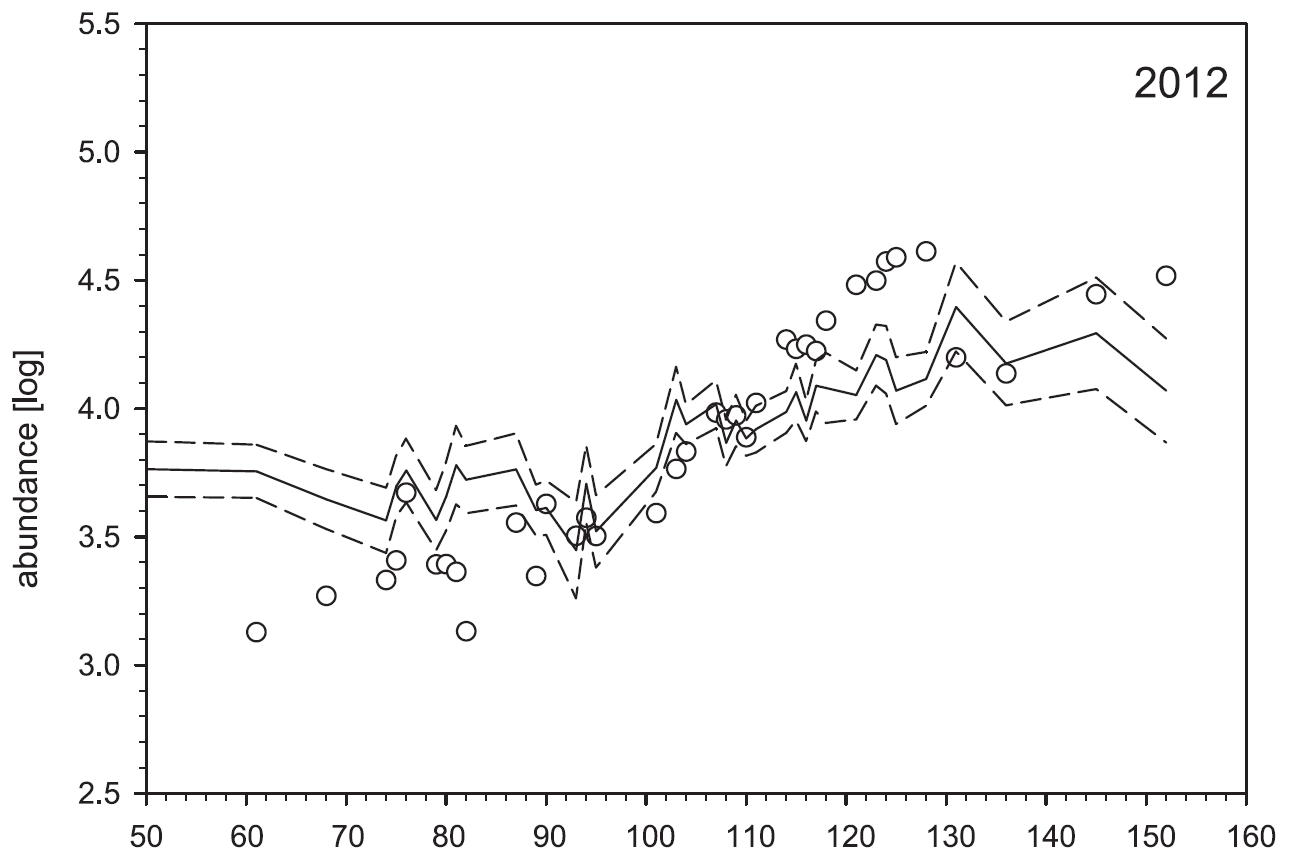


Julian day
