# Supplementary material for: Recurring patterns in bacterioplankton dynamics during coastal spring algae blooms
Source: eLife. 2016 Apr 7;5:e11888. doi: 10.7554/eLife.11888 (PMC4829426; doi:10.7554/eLife.11888)
Supplement: Supplementary file 8. — Two additional metagenomes are listed in italics for completeness, one from a test run sampled on August 21th, 2008, and a metagenome of particle-attached (3 - 10 µm) bacterioplankton sampled on April 14th, 2009. DOI: http://dx.doi.org/10.7554/eLife.11888.018 [file elife-11888-supp8.docx]

| date (yyyy/mm/dd) | sequencing platform | 454 PTPs or Illumina lanes | number of reads | raw bases [bp] | assembled bases [bp] | accession numbers of raw data |
| --- | --- | --- | --- | --- | --- | --- |
| *2008/08/21* | *454 FLX Ti* | *0.5* | *671,375* | *381,301,323* | *31,532,772* | *ERX069459* |
| 2009/02/11 | 454 FLX Ti | 2.5 | 2,203,446 | 695,124,081 | 49,074,624 | ERX069460 - ERX069464 |
| 2009/03/31 | 454 FLX Ti | 2.5 | 1,666,535 | 572,732,655 | 44,909,033 | ERX069465 - ERX069469 |
| 2009/04/07 | 454 FLX Ti | 2.0 | 2,109,239 | 692,367,290 | 52,739,455 | ERX069470 - ERX069473 |
| 2009/04/14 | 454 FLX Ti | 4.0 | 4,588,441 | 1,379,926,332 | 96,006,654 | ERX069474 - ERX069481 |
| *2009/04/14* | *454 FLX Ti* | *2.0* | *2,114,794* | *462,049,389* | *19,769,747* | *ERX069482 - ERX069485* |
| 2009/06/16 | 454 FLX Ti | 1.0 | 1,120,072 | 379,893,755 | 29,795,023 | ERX069490 - ERX069491 |
| 2009/09/01 | 454 FLX Ti | 2.0 | 2,714,430 | 1,106,130,650 | 79,162,758 | ERX069492 - ERX069495 |
| 2010/03/03 | Illumina HiSeq 2000 | 1.0 | 285,509,546 | 42,826,431,900 | 537,254,816 | SRA212914 |
| 2010/04/08 | Illumina HiSeq 2000 | 1.0 | 330,603,192 | 49,590,478,800 | 315,764,252 | SRA212908 |
| 2010/05/04 | Illumina HiSeq 2000 | 1.0 | 274,336,674 | 41,150,501,100 | 453,049,862 | SRA212889 |
| 2010/05/18 | Illumina HiSeq 2000 | 1.0 | 309,971,624 | 46,495,743,600 | 512,323,944 | SRA212588 |
| 2011/03/24 | Illumina HiSeq 2000 | 1.0 | 304,742,176 | 45,711,326,400 | 629,053,950 | SRA212575 |
| 2011/04/28 | Illumina HiSeq 2000 | 1.0 | 295,426,382 | 44,313,957,300 | 541,779,407 | SRA212530 |
| 2011/05/26 | Illumina HiSeq 2000 | 1.0 | 324,219,736 | 48,632,960,400 | 603,965,808 | SRA212476 |
| 2012/03/08 | Illumina HiSeq 2000 | 1.0 | 311,415,662 | 46,712,349,300 | 573,812,810 | SRA212475 |
| 2012/04/16 | Illumina HiSeq 2000 | 1.0 | 258,751,220 | 38,812,683,000 | 543,911,422 | SRA212473 |
| 2012/05/10 | Illumina HiSeq 2000 | 1.0 | 272,267,348 | 40,840,102,200 | 614,071,648 | SRA212472 |

**Supplementary file 8.** Statistics of the 16 metagenomes from free-living (0.2 - 3.0 µm) North Sea bacterioplankton that were used in this study. Two additional metagenomes are listed in italics for completeness, one from a test run sampled on August 21^th^, 2008, and a metagenome of particle-attached (3 - 10 µm) bacterioplankton sampled on April 14^th^, 2009.
