# Supplementary material for: Recurring patterns in bacterioplankton dynamics during coastal spring algae blooms
Source: eLife. 2016 Apr 7;5:e11888. doi: 10.7554/eLife.11888 (PMC4829426; doi:10.7554/eLife.11888)
Supplement: Supplementary file 10. — Taxonomic bins with too small sizes for a sound analysis were excluded (red text). DOI: http://dx.doi.org/10.7554/eLife.11888.020 [file elife-11888-supp10.docx]

|  | *Flavobacteriia* | | | | | *Gammaproteobacteria* | |
| --- | --- | --- | --- | --- | --- | --- | --- |
| sample date  [yyyy-mm-dd] | NS5 marine group | NS3a marine group | *Cryomorphaceae* | *Formosa* | *Polaribacter* | *Alteromonadales* | *Reinekea* |
| 2009-02-11 | 28,268 | 25,379 | 184,865 | 35,849 | 101,842 | 1,135,219 | 0 |
| 2009-03-31 | 116,304 | 95,244 | 252,054 | 79,460 | 142,545 | 2,225,525 | 0 |
| 2009-04-07 | 63,682 | 605,646 | 604,238 | 2,650,935 | 2,006,910 | 963,523 | 1,042 |
| 2009-04-14 | 1,134,678 | 1,249,015 | 914,593 | 1,493,263 | 4,792,871 | 4,794,839 | 2,745,166 |
| 2009-06-16 | 14,273 | 2,475 | 153,530 | 7,471 | 80,618 | 469,165 | 0 |
| 2009-09-01 | 8,102 | 1,961 | 92,732 | 630,404 | 125,352 | 618,415 | 0 |
| 2010-03-03 | 2,013,125 | 1,036,865 | 4,085,158 | 56,335 | 4,089,827 | 6,408,574 | 9,082 |
| 2010-04-08 | 2,382,058 | 1,566,909 | 2,257,273 | 310,554 | 16,607,136 | 9,180,453 | 3,012,071 |
| 2010-05-04 | 2,134,135 | 1,469,249 | 3,239,012 | 9,234,087 | 24,302,119 | 13,178,107 | 3,140,938 |
| 2010-05-18 | 2,137,903 | 1,189,810 | 6,704,274 | 8,843,004 | 23,486,022 | 7,853,842 | 3,100,614 |
| 2011-03-24 | 2,273,661 | 1,398,359 | 8,046,122 | 54,301 | 786,007 | 5,441,592 | 3,640 |
| 2011-04-28 | 2,519,249 | 1,564,862 | 13,839,867 | 343,447 | 10,450,433 | 5,518,637 | 2,184 |
| 2011-05-26 | 2,660,838 | 1,554,456 | 6,750,665 | 5,032,885 | 8,112,484 | 8,919,589 | 14,981 |
| 2012-03-08 | 2,039,920 | 1,399,610 | 4,076,879 | 58,269 | 2,248,308 | 5,105,609 | 1,935 |
| 2012-04-16 | 2,360,980 | 1,573,626 | 10,561,810 | 64,587 | 3,403,386 | 3,859,935 | 533 |
| 2012-05-10 | 2,234,119 | 1,323,847 | 13,158,017 | 3,875,459 | 9,246,259 | 6,076,873 | 3,029 |

**Supplementary file 10.** Sizes in basepairs of the metagenome taxonomic bins that were used for CAZyme frequency analyses on order to genus level. Taxonomic bins with too small sizes for a sound analysis were excluded (red text).
