# Supplementary material for: Recurring patterns in bacterioplankton dynamics during coastal spring algae blooms
Source: eLife. 2016 Apr 7;5:e11888. doi: 10.7554/eLife.11888 (PMC4829426; doi:10.7554/eLife.11888)
Supplement: Supplementary file 11. — Searches were performed against the CAZy database, the dbCAN database and the Pfam database using E-value thresholds that were adjusted for each family by extensive manual annotations. CAZymes were only annotated when at least two of the three database searches yielded positive results (GH = glycoside hydrolase; CBM = carbohydrate- binding module; CE = carbohydrate esterase; PL = polysaccharide lyase; GT = glycoside transferase; AA = auxiliary activities). DOI: http://dx.doi.org/10.7554/eLife.11888.021 [file elife-11888-supp11.docx]

| **CAZyme family** | **E-value** |
| --- | --- |
| CAZy database searches (BLAST) | |
| GH4, GH10, GH17, GH18, GH30, GH31, GH43, GH92 | 1E-40 |
| GH5, GH13, GH16, GH23, GH29 | 1E-35 |
| all other GH families | 1E-30 |
| all CBM families | 1E-30 |
| all CE families | 1E-30 |
| PL7 | 1E-40 |
| all other PL families | 1E-30 |
| all GT families | 1E-30 |
| all AA families | 1E-30 |
| dbCAN database searches (HMMER) | |
| GH42, GH73, GH92 | 1E-10 |
| all other GH families | 1E-05 |
| CBM4, CBM6, CBM11, CBM20, CBM22, CBM35, CBM48, CBM50, CBM67 | 1E-03 |
| all other CBM families | 1E-05 |
| all CE families | 1E-05 |
| all PL families | 1E-05 |
| all GT families | 1E-05 |
| all AA families | 1E-05 |
| Pfam database searches (HMMER) | |
| all GH families | 1E-05 |
| all CBM families | 1E-05 |
| all CE families | 1E-05 |
| all PL families | 1E-05 |
| all GT families | 1E-05 |
| all AA families | 1E-05 |

**Supplementary file 11.** E-value thresholds used for automated CAZyme family detection. Searches were performed against the CAZy database, the dbCAN database and the Pfam database using E-value thresholds that were adjusted for each family by extensive manual annotations. CAZymes were only annotated when at least two of the three database searches yielded positive results (GH = glycoside hydrolase; CBM = carbohydrate- binding module; CE = carbohydrate esterase; PL = polysaccharide lyase; GT = glycoside transferase; AA = auxiliary activities).
